# Best Practices in Constant pH MD Simulations: Accuracy and Sampling

**DOI:** 10.1021/acs.jctc.2c00517

**Published:** 2022-09-15

**Authors:** Pavel Buslaev, Noora Aho, Anton Jansen, Paul Bauer, Berk Hess, Gerrit Groenhof

**Affiliations:** † Nanoscience Center and Department of Chemistry, University of Jyväskylä, 40014 Jyväskylä, Finland; ‡ Department of Applied Physics, Science for Life Laboratory, KTH Royal Institute of Technology, 100 44 Stockholm, Sweden; ⊥ Department of Applied Physics and Swedish e-Science Research Center, Science for Life Laboratory, KTH Royal Institute of Technology, 100 44 Stockholm, Sweden

## Abstract

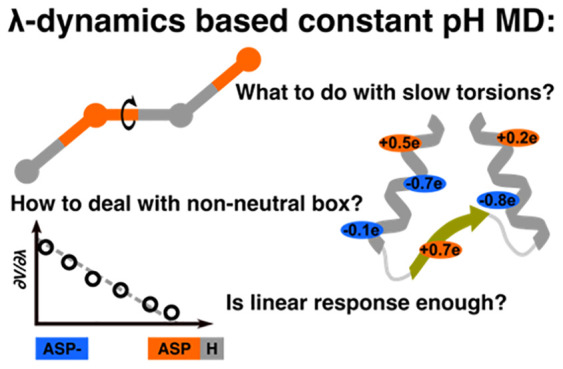

Various approaches have been proposed to include the effect of pH in molecular dynamics (MD) simulations. Among these, the λ-dynamics approach proposed by Brooks and co-workers [Kong, X.; Brooks III, C. L. *J. Chem. Phys.***1996**, **105**, 2414−2423] can be performed with little computational overhead and hfor each typeence be used to routinely perform MD simulations at microsecond time scales, as shown in the accompanying paper [Aho, N. et al. *J. Chem. Theory Comput.***2022**, DOI: 10.1021/acs.jctc.2c00516]. At such time scales, however, the accuracy of the molecular mechanics force field and the parametrization becomes critical. Here, we address these issues and provide the community with guidelines on how to set up and perform long time scale constant pH MD simulations. We found that barriers associated with the torsions of side chains in the CHARMM36m force field are too high for reaching convergence in constant pH MD simulations on microsecond time scales. To avoid the high computational cost of extending the sampling, we propose small modifications to the force field to selectively reduce the torsional barriers. We demonstrate that with such modifications we obtain converged distributions of both protonation and torsional degrees of freedom and hence consistent p*K*_a_ estimates, while the sampling of the overall configurational space accessible to proteins is unaffected as compared to normal MD simulations. We also show that the results of constant pH MD depend on the accuracy of the correction potentials. While these potentials are typically obtained by fitting a low-order polynomial to calculated free energy profiles, we find that higher order fits are essential to provide accurate and consistent results. By resolving problems in accuracy and sampling, the work described in this and the accompanying paper paves the way to the widespread application of constant pH MD beyond p*K*_a_ prediction.

## Introduction

Thanks to improvements in algorithms, force fields, and computer hardware, molecular dynamics (MD) simulations have become a versatile tool for investigating the conformational landscape of complex biomolecular systems at the atomic level.^1−5^ An important algorithmic improvement has been the explicit inclusion of pH in MD simulations,^6−12^ as pH is an important experimental parameter that affects the structure and dynamics of biomolecules. To provide the users of the GROMACS MD package^13^ with access to simulations at constant pH, we have implemented the λ-dynamics-based constant pH approach by Brooks and co-workers.^9,14^ In contrast to the previous implementation in a fork of GROMACS 3.3,^10^ the new implementation, which is described in an accompanying paper,^15^ is efficient and constant pH MD simulations can be performed with about 25% for CPU, 30−40% for CPU + GPU computational overhead compared to normal MD simulations irrespective of the number of titratable sites in the system. In the accompanying methodological paper,^15^ we also demonstrate that the method can be successfully applied to calculate p*K*_a_ values of titratable sites in a protein. The purpose of this paper is to provide users with guidelines and recommendations on how to set up and perform constant pH MD simulations, including the necessary parametrization steps.

In constant pH MD simulations, titratable groups can dynamically change their protonation state. These changes are driven by interactions between the group and the chemical environment (modeled with a force field) and the aqueous proton concentration (modeled with a pH potential). Because at the force field level a number of contributions to the free energy of (de)protonation are not included explicitly, i.e., quantum mechanical interactions associated with bond breakage and formation as well as the actual proton particle, corrections to the force field are needed in λ-dynamics-based constant pH MD. In GROMACS, these corrections are implemented as analytical functions, V^MM^(λ_*j*_), fitted to the free energy profile associated with the deprotonation of a titratable residue *j* at the force field level.^15^

The accuracy of such free energy profiles depends not only on how closely the force field model represents the true potential energy surface but also on the convergence of sampling of all other degrees of freedom in the system. Therefore, whereas in normal MD the accuracy of the dynamics depends solely on the quality of the force field, the accuracy of λ-dynamics-based constant pH MD depends additionally on whether all relevant degrees of freedom are sampled sufficiently in the simulations required for parametrizing the correction potentials.

We found that insufficient sampling of the dihedral degrees of freedom in the amino acid side chains can lead to poor convergence in the deprotonation free energy profiles, as also observed by Klimovich and Mobley in simulations without constant pH.^16^ We traced the lack of the dihedral sampling to the barriers that separate the minima in the torsion potentials. These barriers are too high to reach a converged sampling of the dihedral free energy landscape on the time scales of typical constant pH MD simulations. Because the interaction between the titratable group and the environment depends critically on the dihedral angles of the side chain, a lack of convergence in these dihedral angles also affects the sampling of the protonation states.

Rather than increasing the time scale of the MD simulations to obtain converged dihedral and protonation state distributions or introducing enhanced sampling techniques,^12,17−21^ we propose reducing the barriers for dihedral rotations in a systematic way. We will demonstrate that such optimized dihedral force field parameters improve p*K*_a_ estimates of amino acids without compromising the overall conformational sampling of the protein.

With a higher accuracy of the underlying deprotonation free energy profiles, we found that the correct sampling of protonation states also depends critically on the order of the polynomial fit used to obtain an analytical form for the correction potential. We show that the commonly accepted first-order fit,^12^ although firmly based on linear response theory,^22^ is not sufficiently accurate and can lead to erroneous protonation dynamics in constant pH MD simulations.

Because the dominant energetic contribution to proton affinity comes from electrostatic interactions,^22^ it is of utmost importance to use an accurate description of such interactions. Constant pH MD simulations have been performed with various electrostatic models, including generalized Born,^9^ shifted cutoff,^23^ and particle mesh Ewald (PME).^10,12^ Of these methods, the Ewald summation-based PME method^24,25^ is generally considered to provide the most accurate description of the electrostatic interactions in periodic biomolecular systems.^26^ Because Ewald summation can only provide accurate results if the simulation box remains neutral,^27^ the charge fluctuations associated with the dynamic protonation and deprotonation in constant pH MD simulations need to be compensated to prevent artifacts. Titratable sites can be directly coupled to special particles, modeled as ions or water molecules,^21,28^ such that charge is transferred directly between the titratable site and that particle. Alternatively, all sites can be coupled collectively to a sufficiently large number of buffer particles.^29^ The latter approach has the advantage that spontaneous fluctuations in the interaction of the buffer particles with their environment affect all titratable sites to the same extent. The disadvantage is that the setup and parametrization of the buffer approach are more involved, as these require selecting the number of buffers and parametrizing their interaction with the rest of the system. To facilitate the use of buffers in constant pH MD, we provide a parametrization strategy aimed at preventing buffer clustering, buffer binding to titratable sites, and buffer permeation into hydrophobic regions. We demonstrate that buffers parametrized with this strategy also avoid finite-size effects associated with the periodicity of small simulation boxes.^30,31^

## Methods

Here, we go through all of the important aspects of the simulation setups.

### Simulated Systems

We performed standard and constant pH MD simulations of the systems listed in Table 1. The original and modified (described in detail below) CHARMM36m^32,33^ force fields were used in all simulations. The table also presents the box size, the number of CHARMM36 TIP3P water molecules,^34−36^ the number of ions, and buffer particles included in each system. The fitting coefficients of the V^MM^ correction potential for the buffer particles were obtained with system BUF_1_. To find the optimal charge range and Lennard–Jones parameters for the buffer particles, enhanced sampling simulations with the accelerated weight histogram (AWH) method were performed on system BUF_2_. Systems ADA, AEA, AKA, and AHA are alanine tripeptides with capped termini and as the central residue aspartic, glutamic, lysine, and histidine amino acids, respectively. AAA_1_ and AAA_2_ systems are alanine tripeptides with protonated termini. C- and N-termini were made titratable in AAA_1_ and AAA_2_ systems, respectively. Two sets of simulations of the cardiotoxin V protein were performed. System 1CVO_1_ was used to calculate the p*K*_a_ values of titratable residues, while the larger system 1CVO_2_ was used to compute the radial distribution function of the buffer particles around the protein. The membrane systems MEMB_1_ and MEMB_2_ contained 106 1-palmitoyl-2-oleoyl-glycero-3-phosphocholine (POPC) lipids. The starting coordinates and topologies of all systems are provided as Supporting Information.

**Table 1:** Table of Simulated Systems^a^

system	box size (nm^3^)	no. of waters	no. of ions	force field
BUF_1_	5 × 5 × 5	∼4000	11 Na, 11 Cl, 2 Buf	CHARMM36m
BUF_2_	3 × 3 × 3	∼3000	1 or 2 Buf	CHARMM36m
ADA	5 × 5 × 5	∼4000	11 Na, 11 Cl, 1 or 10 Buf	CHARMM36m, CHARMM36m-cph
ADA_3_	3 × 3 × 3	∼850	2 Na, 2 Cl, 10 Buf	CHARMM36m-cph
ADA_7_	7 × 7 × 7	∼11100	31 Na, 31 Cl, 10 Buf	CHARMM36m-cph
ADA_low salt_	5 × 5 × 5	∼4000	4 Na, 4 Cl, 10 Buf	CHARMM36m-cph
ADA_high salt_	5 × 5 × 5	∼4000	38 Na, 38 Cl, 10 Buf	CHARMM36m-cph
AEA	5 × 5 × 5	∼4000	11 Na, 11 Cl, 1 or 10 Buf	CHARMM36m, CHARMM36m-cph
AKA	5 × 5 × 5	∼4000	11 Na, 12 Cl, 1 or 10 Buf	CHARMM36m, CHARMM36m-cph
AHA	5 × 5 × 5	∼4000	11 Na, 12 Cl, 1 or 10 Buf	CHARMM36m, CHARMM36m-cph
AAA_1_	5 × 5 × 5	∼4000	11 Na, 12 Cl, 1 or 10 Buf	CHARMM36m, CHARMM36m-cph
AAA_2_	5 × 5 × 5	∼4000	11 Na, 11 Cl, 1 or 10 Buf	CHARMM36m, CHARMM36m-cph
1CVO_1_	6.8 × 7.8 × 7.1	∼12400	35 Na, 48 Cl, 20 Buf	CHARMM36m, CHARMM36m-cph
1CVO_2_	7.1 × 7.1 × 7.1	∼11400	13 Cl, 150 Buf	CHARMM36m
MEMB_1_	5.9 × 5.9 × 8.1	∼4200	8 Na, 8 Cl, 50 Buf	CHARMM36m
MEMB_2_	5.9 × 5.9 × 8.1	∼4200	8 Na, 8 Cl, 1 Buf	CHARMM36m
SOL	4.3 × 4.3 × 4.3	∼2600	50 Buf	CHARMM36m

a We denote the modified CHARMM36m force field as CHARMM36m-cph.

### Simulation Setup

Periodic boundary conditions were applied in all systems. Electrostatic interactions were modeled with the particle mesh Ewald method,^24,25^ while van der Waals interactions were modeled with Lennard–Jones potentials which were smoothly switched to zero in the range from 1.0 to 1.2 nm. Simulations were performed at a constant temperature of 300 K, maintained by the v-rescale thermostat,^37^ with a time constant of 0.5 ps^–1^ and at a constant pressure of 1 bar, maintained by the Parrinello–Rahman barostat,^38^ with a period of 2.0 ps. The leapfrog integrator with an integration step of 2 fs was used. Bond lengths to hydrogens in the solute were constrained with the LINCS algorithm,^39^ while the internal degrees of the CHARMM TIP3P water molecules^35,36^ were constrained with the SETTLE algorithm.^40^ Prior to the constant pH MD simulations, the energy of all systems was minimized using the steepest descent method, followed by a 1 ns equilibration.

### Constant pH MD Simulation Setup

In the constant pH MD simulations, the mass of λ-particles was set to 5 atomic units and the temperature was kept constant at 300 K with a separate v-rescale thermostat for the λ degrees of freedom^37^ with a time constant of 2.0 ps^–1^. The single-site representation, defined and described in the accompanying paper,^15^ was used for Asp, Glu, Lys, C-ter, and N-ter, whereas the multisite representation, also described in that paper, was used for His.^15,41,42^ The multisite representation models each physical state of groups with chemically coupled titratable sites with an independent λ-coordinate and takes chemical coupling into account by applying the linear constraint on these λ-coordinates requiring .^15^ The same pH and biasing potentials were used as in Aho et al.^15^ In the sampling simulations of single titratable residues, the pH was set equal to the p*K*_a_ and the barrier height of the biasing potential was set to zero. The titration of the cardiotoxin V (PDB ID 1CVO(43)) protein was performed by running 10 independent replicas of 100 ns each for 15 equidistantly spaced pH values in the range from 1.0 to 8.0 using both the original and a modified CHARMM36m force field. In the titration simulations, the barrier height of the biasing potential was set to 7.5 kJ mol^–1^ for groups modeled with a single-site representation and to 5 kJ mol^–1^ for groups modeled with a multisite representation.

### Reference Simulations

The constant pH simulations require a correction potential *V*^MM^(λ_*j*_) for each titratable residue *j*. These correction potentials are the integrals of polynomial fits to the expectation value of ⟨*∂V*/*∂λ*⟩_λ_ in reference state simulations at fixed λ-values.^15^ Thus, after integration, an *n*th-order polynomial fit to ⟨*∂V*/*∂λ*⟩_λ_ yields an (*n* + 1)th-order polynomial function that represents *V*^MM^(λ). However, in our implementation, the fit to ⟨*∂V*/*∂λ*⟩_λ_ was used, rather than *V*^MM^(λ). We thus refer to the fitting order as the order of the polynomial fit to ⟨*∂V*/*∂λ*⟩_λ_.

We performed the reference simulations as follows: The partial charges in the tripeptide systems were linearly interpolated between λ = −0.1 and λ = 1.1 with a step of 0.05. For His, all three λ coordinates were changed under the constraint λ_1_ + λ_2_ + λ_3_ = 1. For each set of λ-values, called a grid point, we performed an 11 ns MD simulation in which the *∂V*/*∂λ*_*j*_ were saved every picosecond and accumulated. The total charge of the system was kept neutral by simultaneously changing the charge of a single buffer particle. The fitting procedure is described in full detail in the accompanying paper.^15^

### Dihedral Free Energy Profiles

Because the sampling of protonation states is tightly coupled to the sampling of side chain dihedral degrees of freedom, we computed the free energy profiles associated with the rotation of the dihedrals in the side chain of the central amino acid in the capped tripeptide systems (Table 1) by means of umbrella sampling.^44,45^ As the first step, we performed 20 ns MD simulations with a time-dependent potential on the dihedral angle with a force constant of 418.4 kJ mol^–1^ rad^–2^. The center of this potential was moved from 0° to 360° with a rate of 18° ns^–1^. From these simulations, frames with dihedral angles closest to 0°, 10°, 20°, etc., were selected as references for the umbrella replicas. The difference between the dihedral angle in the selected frames and the target angle was always below 0.1°. Then, we performed 36 umbrella sampling simulations of 11 ns with a harmonic restraining potential centered at the reference dihedral angle and a force constant of 418.4 kJ mol^–1^ rad^–2^. We used the WHAM procedure,^46^ implemented in GROMACS,^47^ to unbias these umbrellas and obtain free energy profiles associated with the full rotation of the dihedral angle.

### Dihedral Potential Energy Profiles at the QM and MM Levels

To check the validity of the proposed force field modifications, we computed the potential energy profiles for the N–C_α_–C_β_–C_γ_ dihedral of aspartic acid with capped residues. The profiles were computed at both quantum mechanical (QM) and molecular mechanical (MM) levels. The QM profiles were computed at the MP2/6-31+G* level of theory using the Firefly QC package,^48^ which is partially based on the GAMESS (US)^49^ source code. The MM profiles were computed for both the original and the modified CHARMM36m force fields. The potential energy was computed for the N–C_α_–C_β_–C_γ_ dihedral angle with 10° increments. For each dihedral value, the structures were energy minimized prior to potential energy calculation.

### Accelerated Weight Histogram Alchemical Simulations

Buffer particles are used in constant pH MD to maintain the neutrality of the simulated system. Ideally, buffers should not introduce any artifacts due to binding to titratable groups, binding to each other, or penetrating into hydrophobic regions. To prevent such behavior, we optimized the charge range and Lennard–Jones parameters of the buffers. To this end, we performed a series of enhanced sampling simulations with the accelerated weight histogram method (AWH).^50,51^ In one set of simulations with two buffers in the simulation box (BUF_2_, Table 1), we quantified the sampling efficiency from the friction metric^50,51^ as a function of the absolute charge per buffer particle. In these simulations, the charge of one buffer was changed from 0 to +0.8 while simultaneously the charge of the other buffer was changed from 0 to −0.8 in order to maintain neutrality. The CHARMM36m Lennard–Jones parameters for sodium were used for the buffers in these simulations. In the other set of simulations, we computed the free energy difference between introducing a neutral buffer in water (BUF_2_) and inside the hydrophobic region of a POPC bilayer system (MEMB_2_, Table 1) for various values of the Lennard–Jones parameters of the buffer. In these simulations, the Lennard–Jones interactions between the buffer and the rest of the system were increased from noninteracting at λ = 0 to fully interacting (λ = 1) in 10 discrete steps. For all systems, we simulated 10 replicas of 10 ns, from which the free energy differences and friction coefficients were obtained as averages over the replicas.

### Dihedral Analysis

Distributions of side chain dihedral angles in proteins were derived from publicly shared MD trajectories of proteins with PDB IDs 1U19,^52^2RH1,^53^2Y02,^54^ and 5UEN(55) obtained from the GPCRMD^56^ and SARS-CoV-2 databases (https://covid.molssi.org/).^57^ For each trajectory, the distributions of the following dihedral angles were calculated:

(1) N–C_α_–C_β_–C_γ_ in aspartic acid
(2) N–C_α_–C_β_–C_γ_ in glutamic acid
(3) C_α_–C_β_–C_γ_–C_δ_ in glutamic acid
(4) N–C_α_–C_β_–C_γ_ in histidine
(5) H–O_ϵ2_–C–O_ϵ1_ in aspartic and glutamic acids.

In this work, we also computed the distributions of these dihedrals from standard MD trajectories of cardiotoxin V (PDB ID 1CVO(43)).

### Comparisons of Distributions

To compare two distributions *P*_*i*_(*x*) and *P*_*j*_(*x*), with the corresponding cumulative distribution functions *F*_*i*_(*x*) and *F*_*j*_(*x*), we used Kolmogorov–Smirnov statistics (KSS)^58^
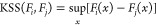
1The larger the KSS, the less similar the distributions are. The distributions were considered consistent when KSS(*F*_*i*_, *F*_*j*_) < 0.03. The KSS was computed using the script from the PCAlipids package.^59,60^

### Titration

To estimate the p*K*_a_ values of titratable groups from multiple simulations at various pH values, we computed the average fraction of deprotonated frames (*S*^deprot^) over all replicas at each pH value. For a group with a single titratable site, this average was obtained as
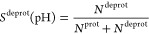
2where *N*^prot^ and *N*^deprot^ are the total number of frames in which the site is protonated and deprotonated, respectively. For titratable sites modeled with the single-site representation, we considered the site protonated if λ is below 0.2 and deprotonated if λ is above 0.8. For sites that are described with the multisite description, we considered a state protonated if the λ associated with the protonated form of the residue is above 0.8 and deprotonated if the λ associated with the deprotonated form of the residue is above 0.8.

To estimate the macroscopic p*K*_a_ value of histidine, which contains two titratable sites N_ϵ_ and N_δ_, we calculated for each pH value the average fraction of frames in which the residue is deprotonated at either of the two sites
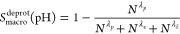
3where , , and  are the number of frames in which λ_*p*_ > 0.8, λ_ϵ_ > 0.8, and λ_δ_ > 0.8.

The averaged fractions at each pH value were fitted to the Henderson–Hasselbalch equation
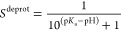
4which yielded the p*K*_a_ values as fitting parameters.

## Results and Discussion

First, we demonstrate that a lack of sampling of the relevant dihedral degrees of freedom in amino acid side chains with the CHARMM36m force field reduces the accuracy of the correction potentials for λ-dynamics. To overcome these convergence problems, we modify the force field by reducing the barriers in the torsion potential and show that this significantly improves the accuracy of the correction potentials and hence the results of constant pH simulations, including p*K*_a_ estimates, without affecting the protein conformational dynamics. After the validation of the modified force field parameters, we show how the buffer particles that maintain the neutrality of the simulation box have to be parametrized to prevent finite-size effects on proton affinities due to periodicity.^30,31^

### Sampling

Klimovic and Mobley have shown that calculated hydration free energies of single amino acids depend on the starting conformation.^16^ Because a few picoseconds typically suffice to sample bond and angle degrees of freedom in the amino acid as well as the rotational degrees of freedom of the water molecules, we speculate that their observation implies a lack of sampling in the dihedral degrees of freedom in the amino acid side chain. Therefore, we systematically analyzed the convergence of both λ-coordinates and dihedral angles during constant pH simulations of single amino acids in water.

We performed 100 ns constant pH MD simulations at pH = p*K*_a_ of systems ADA, AEA, AKA, AHA, AAA_1_, and AAA_2_ (Table 1). To enhance the sampling of the λ-coordinate in these systems, we ran the simulations without a barrier in the biasing potential (*V*^bias^(λ), eq 5 in Aho et al.^15^). The correction potential (*V*^MM^(λ), eq 5 in Aho et al.^15^) was obtained by fitting a third-order polynomial function to the ⟨*∂V*/*∂λ*⟩_λ_ values of the reference trajectories. We will show later that for accurate and reproducible constant pH MD results, a higher order fit is required. Nevertheless, in spite of its limited accuracy, using the same third-order fit for all system suffices to systematically compare the distributions of the relevant degrees of freedom and assess their convergence.

**Figure 1:**
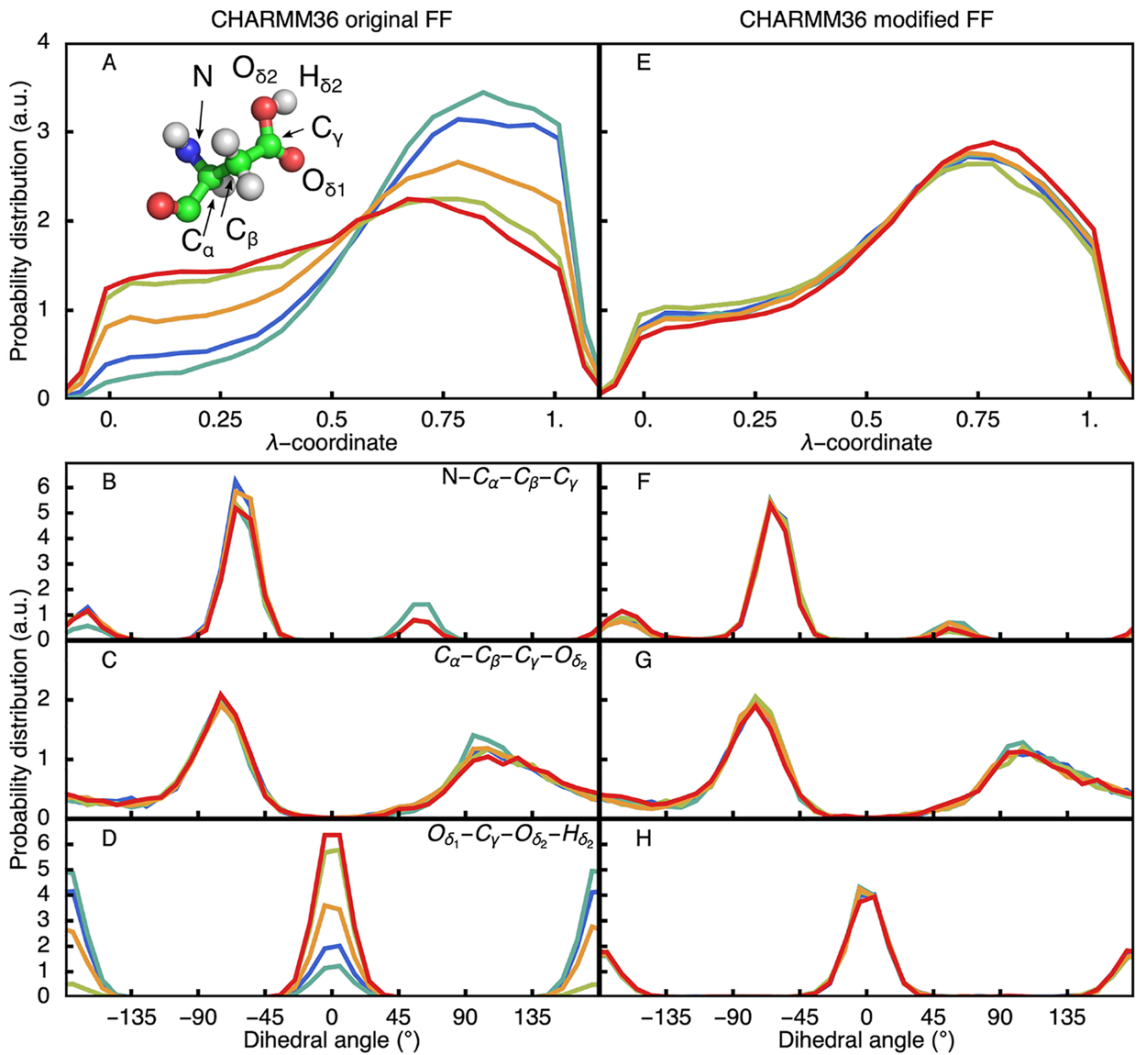
Distributions of the λ-coordinate (A and E) and dihedrals (B–D and F–H) in constant pH MD simulation of Asp with the original (A–D) and modified CHARMM36m (E–H) force field. While the simulations were performed for an ADA tripeptide, only the central aspartic acid is shown for clarity in the inset of A. (A and D) Distributions for third-order fits for ⟨*∂V*/*∂λ*⟩_λ_ obtained with the original force field. Different colors correspond to independent replicas. Distributions for the λ-coordinate (A) as well as distributions for N–C_α_–C_β_–C_γ_ (B) and -C_γ_-- (D) dihedrals are not identical. Right column (E–H) shows the distributions for the modified CHARMM36m force field with third-order fit for ⟨*∂V*/*∂λ*⟩_λ_. Distributions are identical.

In Figure 1A, we show the distributions of the λ-coordinate in five constant pH MD replicas of the ADA system with the original CHARMM36m force field parameters. Distributions of the λ-coordinates in the other systems (AEA, AKA AHA, and AAA_1_ and AAA_2_) are shown in the Supporting Information (SI, Figures S1–S5). The dissimilarity between the λ-distributions in the replicas (maximum KSS between replicas of 0.29, 0.11, 0.04, and 0.095 for ADA, AEA, AHA, and AAA_1_, respectively) indicates a lack of convergence. In addition, the distributions of the dihedral angles, shown in Figure 1B–D, are also not identical for all replicas. Because there is no barrier from the biasing potential for the λ-coordinate, we conclude that the lack of convergence in λ is due to insufficient sampling of the dihedral degrees of freedom.

#### Force Field Modifications

To overcome the lack of sampling, one can increase the simulation time or enhance sampling by means of special algorithms such as replica exchange MD.^61,62^ Replica exchange has been applied frequently in the context of constant pH MD with exchanges between replicas at different temperature (T-REMD), pH (pH-REMD), or both.^20,63−66^ However, because the protonation state has little effect on the torsion barrier height (Figure 3), changing the pH across the pH ladder would do little to enhance the sampling of the dihedral degrees of freedom. Furthermore, REMD methods are computationally more demanding than performing a single MD simulation and also prevent access to the dynamical properties of the system due to the jumps between replicas. As for some applications the dynamical properties may be highly needed, we consider it important that all relevant protonation states can be sampled correctly in a single constant pH MD trajectory. Because the sampling of λ-coordinates is tightly coupled to the sampling of the dihedral angles, convergence within a single trajectory can in principle also be reached by lowering the barriers of the torsion potentials.

Previously, modifications to the force field have been introduced for carboxyl groups. To improve the sampling of the syn and anti conformations of the carboxyl proton in Glu, Asp, and the C-terminus, Brooks and co-workers reduced the barrier for this rotation by a factor of 8 and also scaled the carboxyl oxygen radii by 0.95.^9^ In contrast, Grubmüller and co-workers modified this torsion potential to prevent sampling the anti-conformation altogether.^67^ However, according to our analysis (Figure 1), there is a lack of convergence not only in the carboxyl dihedral angle but also in the other side chain dihedral angles.

**Figure 2:**
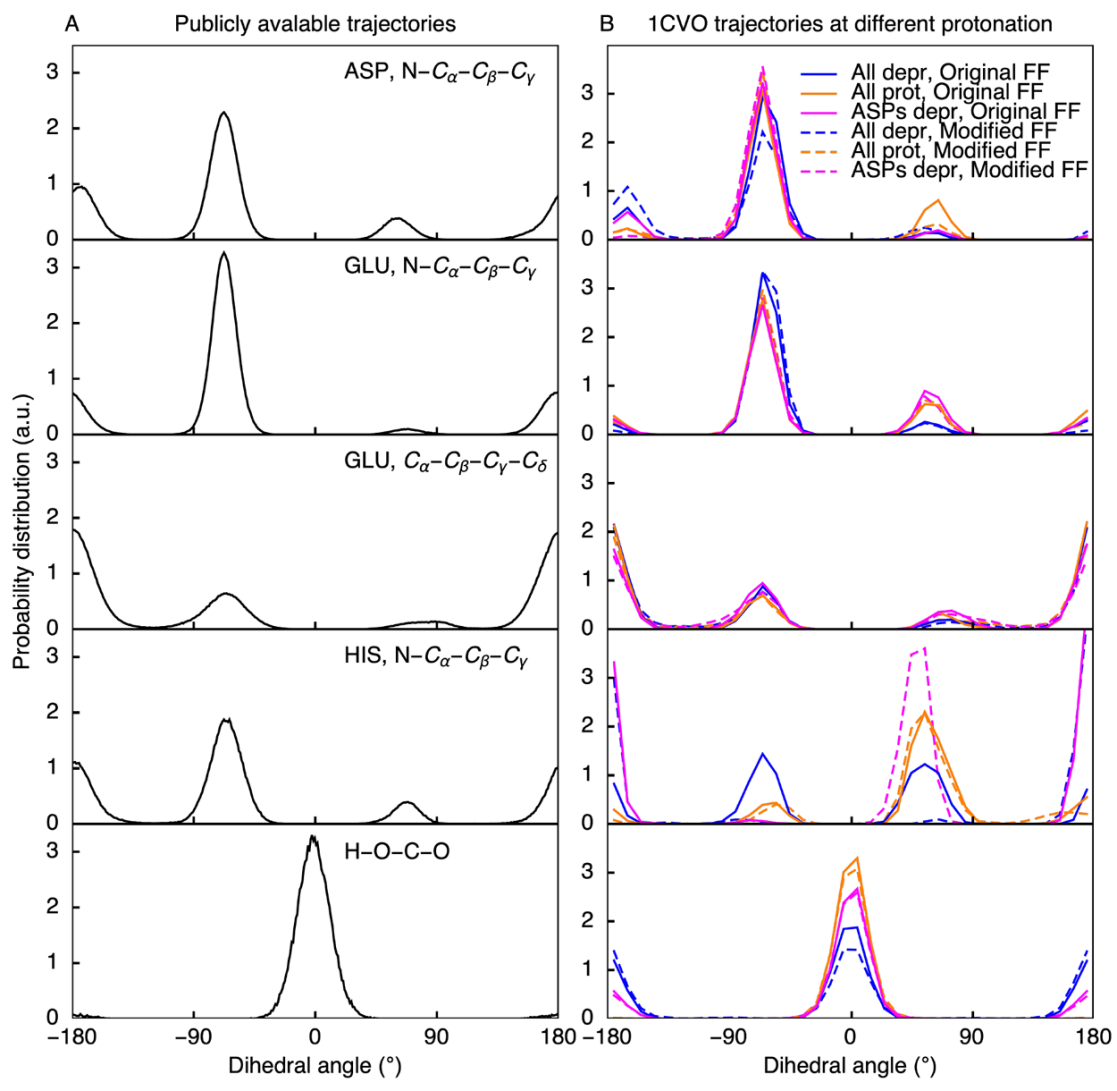
Distributions of dihedral angles for which the torsion potentials were corrected from standard MD simulations. (A) Dihedral distributions from publicly available trajectories^56,57^ (total simulation time ≈ 10 μs). Probability density is significant only around local minima. (B) Dihedral distributions obtained from simulations of cardiotoxin V with both the original and the modified barriers for different protonation states of the titratable residues. Local minima are preserved, and no additional configurations are observed.

Reducing the torsional barriers without affecting the overall sampling of the conformational space is possible only if the regions near such barriers are sparsely sampled. We, therefore, analyzed the distributions of the dihedral angles in the side chains of titratable amino acids in the publicly available trajectories of G-protein-coupled receptors and of SARS-CoV-2 proteins.^56,57^ The distributions of these dihedral angles, plotted in Figure 2A, reflect the shape of the underlying torsion potentials with maxima coinciding with local minima of the potential profiles. The low density near barriers suggests that these barriers are rather high and might be reduced without affecting the dihedral distributions.

To achieve convergence of both dihedral angles and λ-coordinates on a 100 ns time scale, we thus altered the maxima of the torsion potentials by adding a small dihedral angle (ϕ)-dependent correction to the CHARMM36m force field
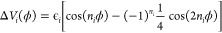
5where *n*_*i*_ is the multiplicity of the torsion angle *i* (i.e., the number of minima) with *n*_*i*_ = 2 for conjugated bonds and *n*_*i*_ = 3 for aliphatic bonds. The parameter ϵ_*i*_ is an empirical coefficient that is optimized such that the barriers are low enough to converge the distribution of ϕ_*i*_ without introducing additional minima on the potential energy surface.

For each side chain dihedral angle of the titratable amino acids, the coefficient ϵ_*i*_ was optimized in an iterative fashion: After an initial guess, we computed the free energy profiles associated with rotation of the dihedral as well as five unbiased 100 ns trajectories at pH = p*K*_a_ with different starting conditions and a biasing potential (*V*^bias^(λ), eq 5 in Aho et al.^15^) without barrier. Prior to these constant pH MD simulations, we recomputed the correction potential, *V*^MM^(λ), by fitting a third-order polynomial to the ⟨*∂V*/*∂λ*⟩_λ_ values obtained from thermodynamic integration simulations performed with the current value of ϵ_*i*_. Free energy profiles were inspected visually for artificial minima, while distributions of both dihedral angles and λ-coordinates were compared between the five unbiased replica runs based on their similarity. The coefficient ϵ_*i*_ was gradually increased until the distributions in the different replicas were sufficiently similar (KSS < 0.03), while at the same time no additional minima appeared in the free energy profiles.

**Figure 3:**
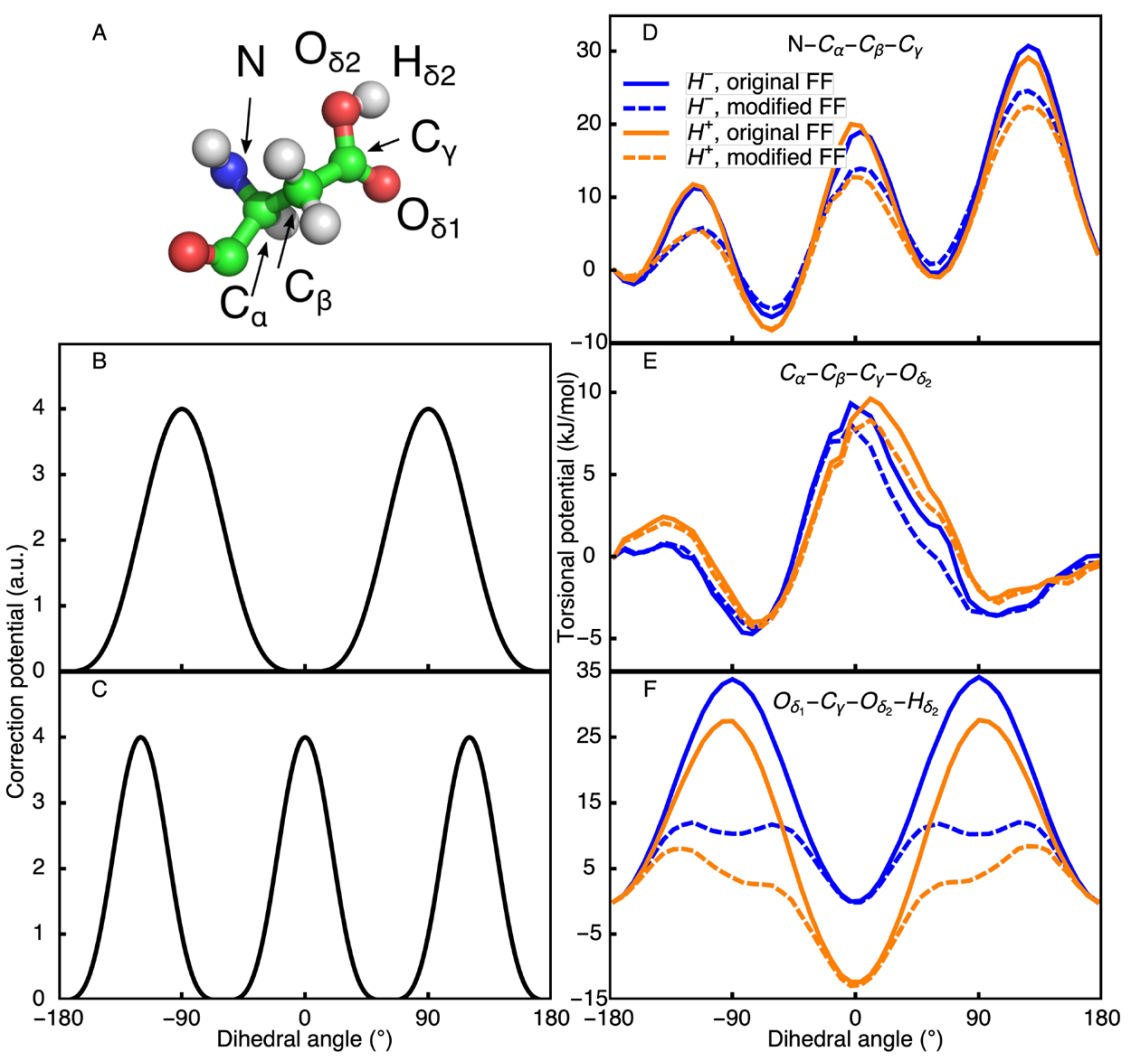
Modification of the torsional barriers in the Asp side chain. (A) Aspartic acid and its atomic nomenclature. (B and C) Corrections added to dihedral torsions of the original force field. Corrections with two (B) and three (C) local minima were used for torsions with two and three local minima, respectively. Heights of the corrections were selected through the iterative process, aimed at achieving consistent λ-distributions without introducing additional local minima in the free energy profiles. (D–F) Original and modified torsional barriers of Asp for both protonated (H^+^) and deprotonated (H^–^) states.

In Figure 3 we show the optimized torsion potentials as well as their effect on the free energy profiles of the dihedral angles in Asp. The corrections and free energy profiles of Glu, His, and the C-terminus are shown in Figures S6–S8 of the SI. With the exception of the C_β_–C_γ_–O_δ_–H dihedral, the corrections introduce no additional minima on the free energy profile of these torsions. Furthermore, as shown on the right panels of Figure 1, the distributions of the dihedrals and λ-coordinates are nearly indistinguishable for all replicas after correction. Because with the corrected potentials the Kolmogorov–Smirnov statistics for Asp, Glu, His, and the C-terminus are 0.028, 0.015, 0.027, and 0.022, we conclude that the corrections improve the convergence of both the λ-coordinates and the dihedral degrees of freedom in constant pH simulations. Note that although the distributions of the λ-coordinate are sufficiently similar, the sampling of these coordinates is not yet uniform (Figure 1E). We will show below that this discrepancy is due to the low order of the polynomial fit used for obtaining the correction potential *V*^MM^(λ).

With the exception of the −C_γ_−–H dihedral, the corrections we propose here lead to changes in the torsion barrier of at most 16 kJ mol^–1^ (i.e., for the Glu N–C_α_–C_β_–C_γ_ torsion). For many biomolecular force fields, the parameters of the torsion potentials are obtained by fitting suitable periodic functions to energies evaluated at the MP2 level of theory.^68−71^ The parameters for each type of torsional potential are simultaneously fitted for multiple amino acids. Therefore, the average root mean squared (RMS) difference between the torsional energy at the CHARMM36m level and that at the MP2 level of theory is on the order of 10 kJ mol^–1^. The RMS deviation between the modified and the original torsion potentials is at most 8 kJ mol^–1^, and the RMS deviation between the ab initio potential at the MP2/6-31+G* level and the N–C_α_–C_β_–C_γ_ torsion potential in ASP is reduced from 4 kJ mol^–1^ for the original CHARMM36m force field to 3.5 kJ mol^–1^ for the modified CHARMM36m force field (Figure S9). Therefore, we conclude that with the corrections of the torsion potentials, the modified force field provides an equally good fit to QM potential profiles as the original CHARMM36m force field.^33,71^

We also performed standard MD simulations and simulated five replicas for 100 ns for the two protonation states of the Asp tripeptide in water using both the original and the modified CHARMM36m force field parameters. Without the modifications, the local minima are not consistently sampled in all replicas (Figure S10). In contrast, with the corrections, identical distributions of the dihedral angles are obtained also in standard MD simulations. In addition, the modifications are essential to sample both syn and anti conformations of the carboxyl proton, in line experiment.^72^ We note, however, that the correction required for sampling both of these conformations significantly alters the shape of the barrier (Figure 3F). Nevertheless, because of their low mass, proton can tunnel through such barriers, and therefore, we consider the shape and height of the torsional barrier less relevant for this specific dihedral than for the other dihedrals.

Finally, we demonstrate that the modifications do not alter the distributions of the dihedral angles in protein simulations. We performed MD simulations of the 1CVO_1_ system both with and without the modifications to the torsion potentials of titratable amino acids with either (i) all of these residues protonated, (ii) all deprotonated, or (iii) all Asp residues deprotonated and all other residues protonated. In Figure 2B, we plot the distributions of the dihedral angles for which corrections were introduced. The high similarity between the distributions suggests that the corrections do not lead to the sampling of different dihedral distributions, even if the relative weights of the minima are slightly altered, in particular, for the H–O–C–O dihedral. We conclude, therefore, that the corrections introduced to facilitate sampling of the dihedral and λ-coordinates do not significantly alter the protein conformational landscape and can hence be used to perform both normal and constant pH MD simulations.

### Quality of the Correction Potential *V*^MM^

#### Reference Potential

To verify that the modified torsion potentials overcome the convergence problems, we performed constant pH simulations at pH = p*K*_a_ and without a barrier in the biasing potential. Because with such a setup the potential energy profile for the λ-particle should be flat, we expected a uniform λ-distribution, provided that the dihedral degrees of freedom are sufficiently sampled. However, as shown in Figure 1E, the distributions are identical between replicas but not uniform despite the corrections to the torsion potentials.

Because both the pH-dependent potential *V*^pH^(λ) and the biasing potential are flat by construction at pH = p*K*_a_, the deviations must originate from discrepancies between the correction potential *V*^MM^(λ) and the underlying free energy profile associated with deprotonation. The correction potential is obtained as a polynomial fit to the ⟨*∂V*/*∂λ*⟩_λ_ values from thermodynamic integration simulations. Because linear response (LR) theory predicts a linear dependence between the hydration free energy and the magnitude of a (point) charge, a first-order fit has often been used to obtain the correction potential for constant pH MD.^9,12^ However, even if the change in the charge dominates the free energy of changing the protonation state, hydrogen-bond rearrangements can contribute as well. Because the effects due to such structural rearrangements are neglected in LR theories, we hypothesized that higher order fits may be necessary for obtaining sufficiently accurate correction potentials.

**Figure 4:**
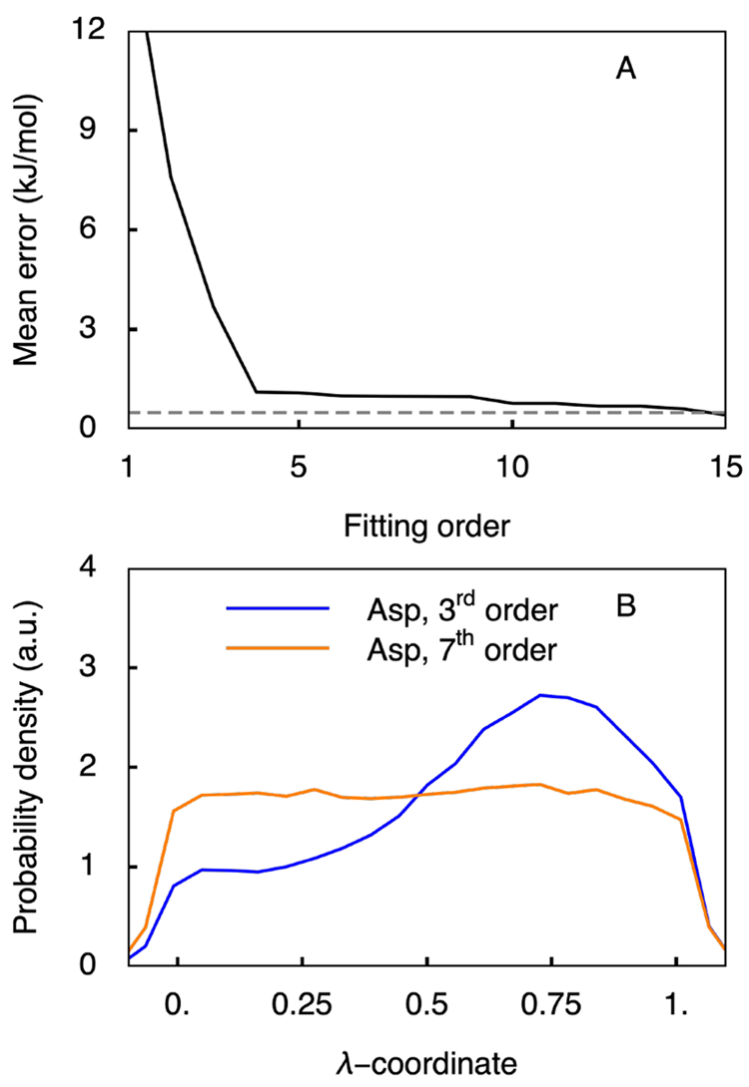
Quality of the *V*^MM^(λ) correction potential as a function of the order of the polynomial fit to ⟨*∂V*/*∂λ*⟩_λ_ for Asp. (A) Fitting error as a function of fitting order (black line). Gray dashed line shows the average error in the calculated ⟨*∂V*^MM^/*∂λ*⟩. (B) Distributions of λ-coordinates for the third- and seventh-order polynomial fits to ⟨*∂V*/*∂λ*⟩_λ_. Whereas with the lower order fit the distribution is significantly rugged, the distribution becomes nearly flat and uniform on the [0, 1] interval of the λ-coordinate if a seventh-order fit is used.

To test our hypothesis, we investigated the accuracy of the polynomial fit to the correction potential. In Figure 4A, we show the mean error of the correction potential with respect to the computed free energy difference associated with deprotonation as a function of the fitting order. For the LR approximation the fitting errors are higher than 10 kJ/mol. In the worst-case scenario, such errors could lead to deviations in predicted p*K*_a_ values of more than one p*K*_a_ unit. With an error of 4 kJ mol^–1^, the third-order fit, used above to address the convergence issues, does not yield a sufficiently accurate representation of the underlying free energy profile. Increasing the order of the polynomial fit reduces this error, and as shown in Figure 4B, at least a seventh-order fit is required to provide a uniform distribution of the λ coordinate for the Asp tripeptide in constant pH MD simulations at pH = p*K*_a_.

Also, for carboxyl groups in the side chains of Glu and in the C-terminus, a polynomial fit to ⟨*∂V*/*∂λ*⟩_λ_ of at least seventh-order is needed to provide a sufficiently accurate correction potential (Figure 4 and Figures S11 and S14 in SI). For the imidazole ring of His with three coupled titratable sites, a seventh-order fit suffices as well (Figure S13), while for the amino bases in the side chain of Lys and the N-terminus, at least an eighth-order fit is required (Figures S12 and S15 in SI). We speculate that the higher order fit is needed for the latter sites due to the larger change in the charge on the central nitrogen atom from −0.3 *e* to −0.96 *e* upon deprotonation. The change in the charge of the carboxylic oxygen from 0.55 *e* to −0.76 *e* is smaller as are the changes on the nitrogen atoms of the imidazole ring of His (from −0.36 *e* to −0.7 *e*).

### Parameterization of Buffer Particles

A change in the protonation state affects the total charge of the simulated system, which can lead to artifacts when Ewald summation is used to treat the electrostatic interactions.^27,31^ In our implementation of constant pH MD, we avoid this problem by introducing titratable buffers into the simulation box that compensate for the charge fluctuations of the titratable residues.^29^

In the original implementation of constant pH MD in GROMACS,^10^ the buffers were hydronium molecules that compensated for the overall charge fluctuations by changing their charge between 0 and +1 *e*. To prevent sampling charges beyond this interval, a biasing potential with steep edges at λ = 0 and 1 was introduced to restrict the range of λ-values. However, this potential still introduces additional forces at the edges of the λ-interval. To avoid the effects of such forces, we use a completely flat biasing potential for the buffers, also outside of the charge interval.

**Figure 5:**
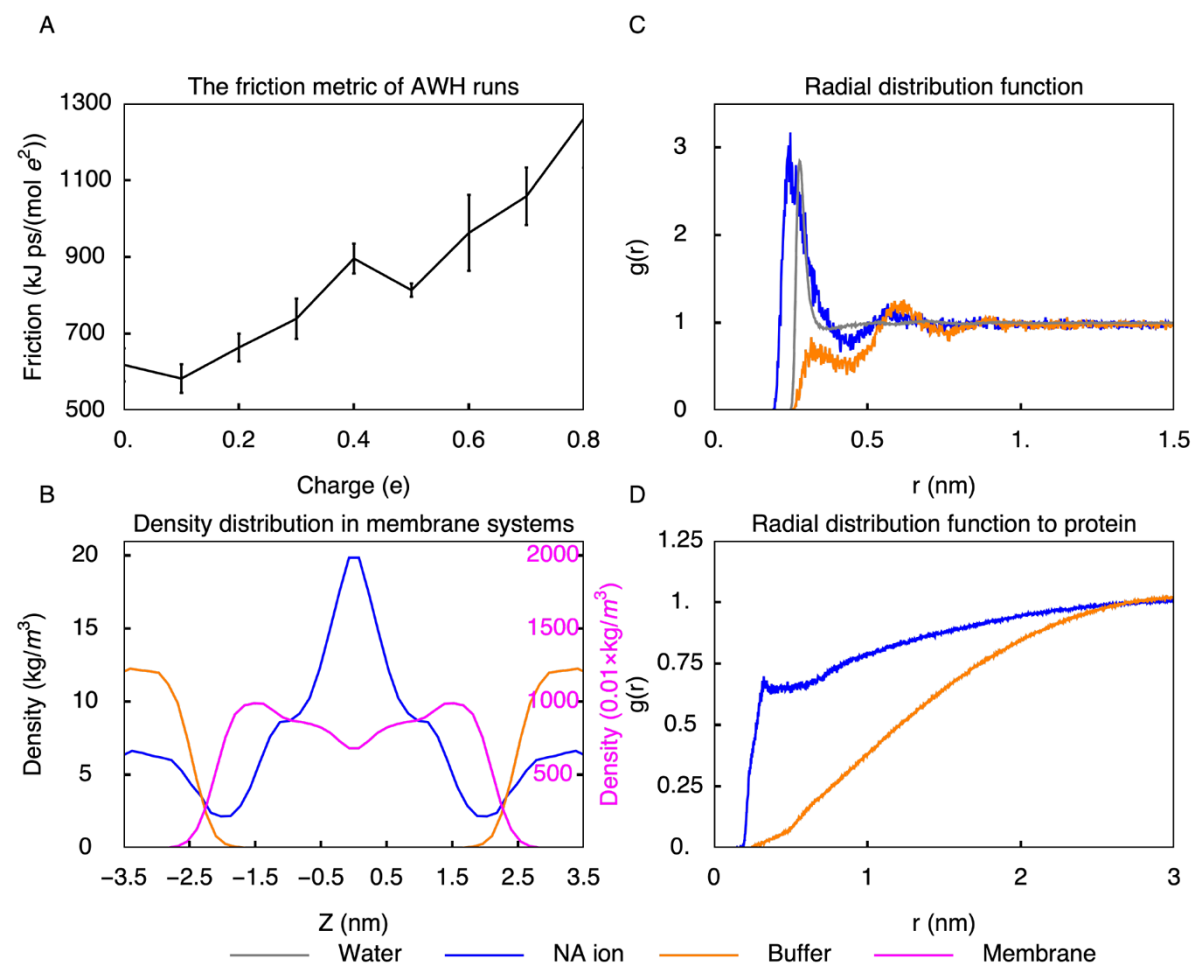
Parameterization of the buffer. (A) AWH friction metric as a function of buffer charge. Since the higher the friction metric is the slower the sampling, the buffers should ideally have low charges. (B) Density distribution of buffers in a membrane system. Optimized buffers do not penetrate into the lipid bilayer, while uncharged sodium ions do. (C) Radial distribution function between buffers. When standard sodium-ion parameters are used as buffers, the tendency to form clusters is high. Optimization of buffer parameters prevents clustering. (D) Radial distribution functions of buffers with respect to the protein. With the original sodium parameters the buffers have a higher tendency to bind to the protein than with the optimized parameters.

Because changing the charge of a buffer particle in solution induces local rearrangements of the hydrogen-bonding network that in turn could affect the proton affinity of a nearby titratable group, we want to minimize the impact of charging the buffer particles. To determine the charge range in which the buffers do not cause significant hydrogen-bond network rearrangement, we ran AWH simulations with two ions, the charges of which are changed simultaneously in opposite directions (BUF_2_ system). From the friction metric available in the AWH method,^50^ we estimated the local diffusion coefficient, which is related to the efficiency of sampling: The higher the friction, the slower the dynamics and the more sampling is required to reach convergence. We calculated the friction coefficient (Figure 5A) for the coordinate associated with changing the charge on the buffer. For charges higher than 0.5 *e*, the friction was more than 50% higher than that for zero charges, reflecting longer correlation times and hence slower dynamics. We, therefore, conclude that the optimal range for the buffer charge is between −0.5 *e* and 0.5 *e*.

The collective λ-coordinate of the buffer particles is not restricted to a fixed interval by a wall-like potential. To avoid the buffer charge exceeding the optimal range, multiple buffer particles are needed in the simulation box. The optimal number of buffers can be calculated based on the analysis of charge fluctuations performed by Donnini et al.^29^

With a small charge, a buffer particle is apolar. To prevent clustering of such apolar particles in water, permeation into hydrophobic areas, such as membrane interiors, or interactions with the protein, the Lennard–Jones parameters (σ and ϵ) of the buffers were chosen such that the buffers have only repulsive interactions with all other atoms, except water. After experimenting with the parameters for the buffer particles, we settled on a σ of 0.25 nm and an ϵ of 4 kJ mol^–1^. This choice leads to decreased clustering of buffers, low buffer concentrations in the proximity of titratable sites, and reduced penetration into hydrophobic regions (Figure 5). The resulting free energies of neutral buffer insertion into water and the hydrophobic region of the membrane are −2.09 ± 0.07 and 1.2 ± 0.6 kJ mol^–1^ compared to 8.45 ± 0.05 and 7.8 ± 0.3 kJ mol^–1^ for insertion of an uncharged sodium ion into these regions.

**Figure 6:**
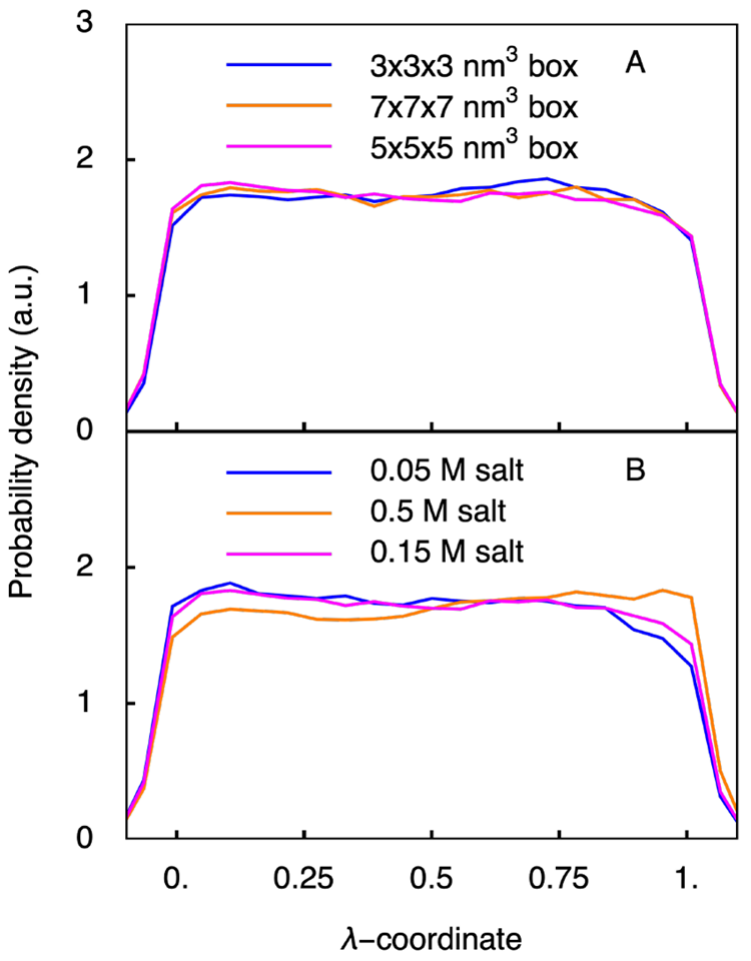
Effect of the box size (A) and ionic strength (B) on the distribution of the λ-coordinate of the ADA systems (ADA, ADA_3_, ADA_7_, ADA_low salt_, ADA_high salt_).

While the primary goal of introducing buffers is to avoid the artifacts associated with a non-neutral periodic simulation box,^27^ there can be other artifacts as well.^30,31^ In particular, the undersolvation caused by solvent orientational restraints due to periodic boundary conditions^30^ could lead to finite-size effects especially for small boxes. To understand if such finite-size effects affect the results of constant pH simulations, we investigated how the distribution of the λ-coordinates depends on the system size. We, therefore, performed constant pH MD simulations for three different box sizes and at various ionic strengths. All simulations were performed at pH = p*K*_a_ and without a barrier in the biasing potential. The uniformity of the distributions in these simulations, shown in Figure 6, suggests that for the box sizes tested, the finite-size effects are negligible.

### Use Case: Consistent Protein Titrations

**Figure 7:**
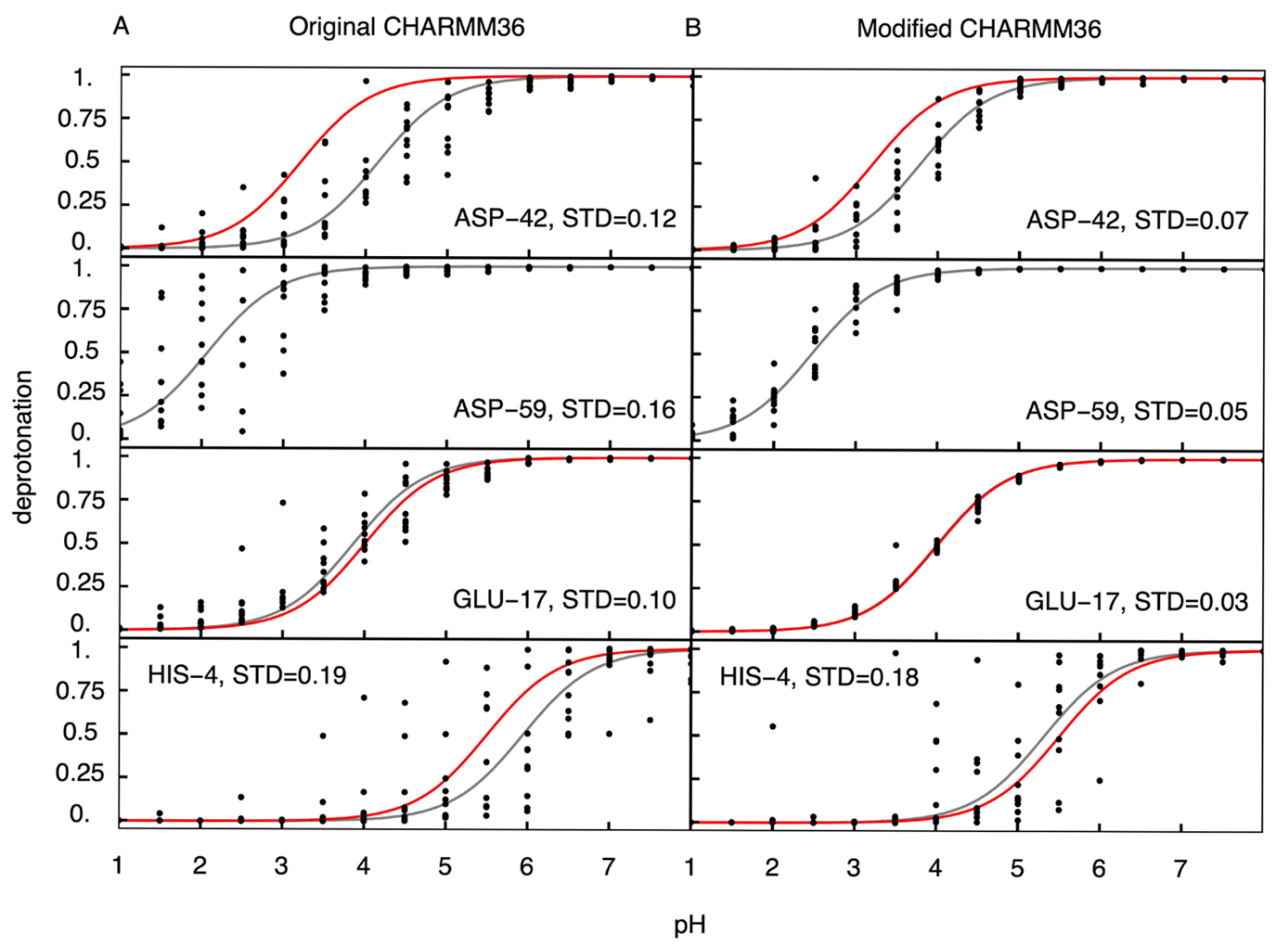
Titration of cardiotoxin V. (A and B) Titration curves of the titratable residues in 1CVO_1_ obtained from constant pH simulations performed with the original and modified CHARMM36 force fields, respectively. Black dots show the deprotonation ratio for the individual replica. Gray lines show the fits to the Henderson–Hasselbalch equation. Red lines are Henderson–Hasselbalch curves computed for the experimental p*K*_a_ value of the corresponding residue.^73,74^ For ASP59, the exact p*K*_a_ is not known. For each curve, the standard deviation between the calculated and the fitted deprotonation ratio, averaged over all pH values and replicas, is shown.

To demonstrate that with the modifications of the torsional barriers, a correction potential obtained by fitting at least a seventh-order polynomial to the ⟨*∂V*/*∂λ*⟩_λ_ values of reference simulations, and buffer particles with optimized parameters, it is possible to perform accurate constant pH MD simulations, we calculated the p*K*_a_ values of all four cardiotoxin V titratable residues. We performed the pH titration simulations with both the original and the modified CHARMM36m force fields. In Figure 7, we show the titration curves obtained in the simulations and compare them to the experiment. Because there is no exact experimental estimate for the p*K*_a_ of ASP59, we only compare the p*K*_a_ values obtained for the other residues. The comparison suggests that the force field corrections improve the p*K*_a_ estimates, but more importantly, the lower deviation between the individual replicas (from 0.12 to 0.07 for ASP42, from 0.16 to 0.05 for ASP59, from 0.1 to 0.03 for GLU17, and from 0.19 to 0.18 for HIS4, Figure 7) suggests that the sampling improves when the modified force field is used.

**Figure 8:**
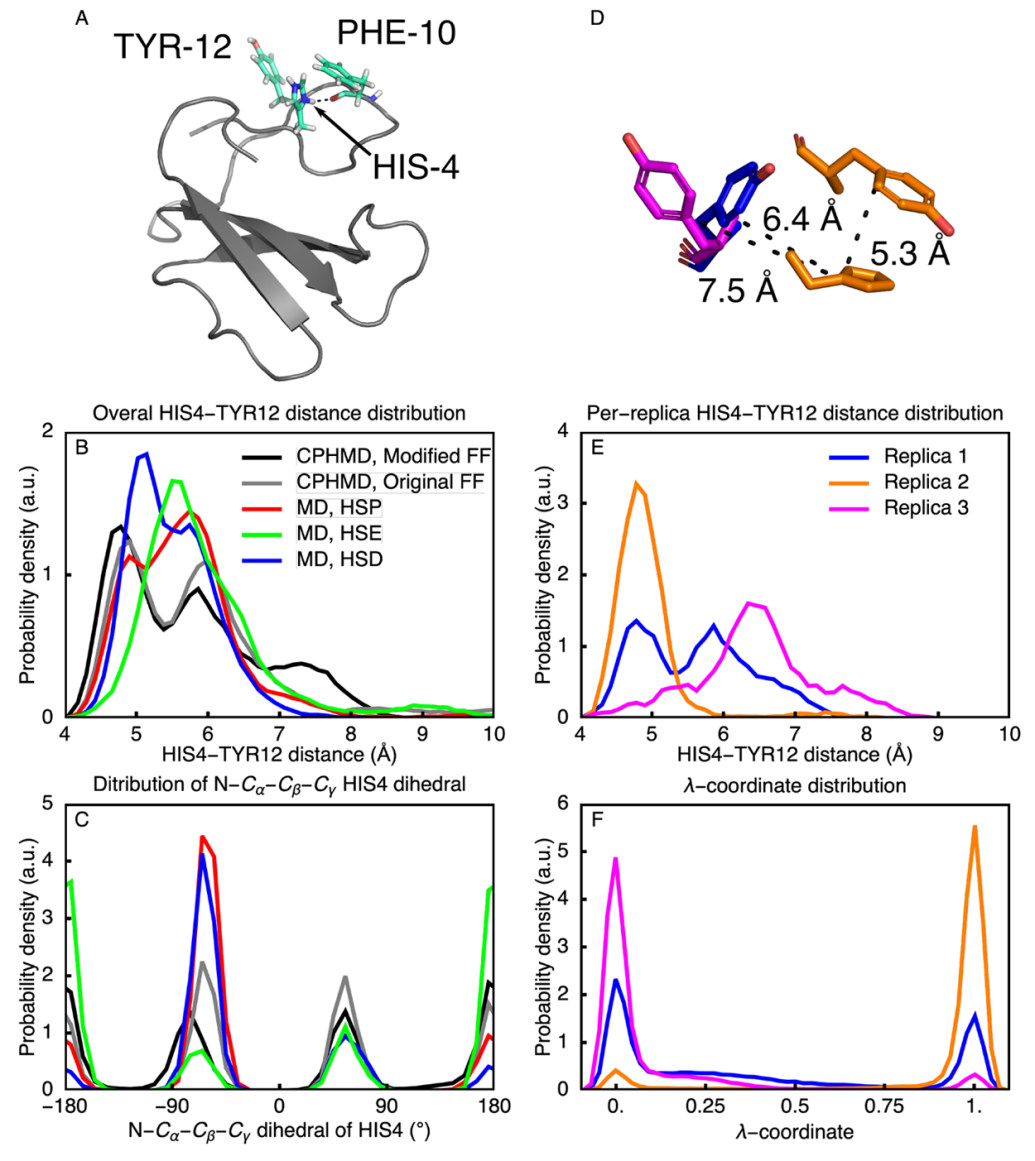
Sampling of cardiotoxin V N-terminal loop. (A) Structure of cardiotoxin V. Protein is shown in cartoon representation with HIS4, PHE10, and TYR12 shown as sticks. (B) Distributions of the distance between the centers of mass of HIS4 and TYR12 for all replicas combined. Combined distributions were calculated for constant pH MD with both the modified and the original force fields as well as for standard MD with HIS4 in all possible protonation states. (C) Distribution of HIS4 N–C_α_–C_β_−C_γ_ torsion. (D) Three observed conformations of TYR12 around HIS4. (E) Distributions of the distance between the centers of mass of HIS4 and TYR12 for individual replicas, computed for constant pH MD with the modified force field. (F) λ distributions of HIS4 protonated state for individual replicas of cardiotoxin V from the constant pH simulations with the modified force field.

Whereas for Asp and Glu the individual titration replicas are consistent when the modified force field is used, the replicas for HIS4 are not converged. As shown in Figure 8A, HIS4 interacts with TYR12 and PHE10. At pH = 5.5, close to the experimental p*K*_a_ of HIS4, we find three dominant conformations of HIS4-TYR12 pair in both normal and constant pH MD trajectories: A close contact (around 5 Å), a medium-range contact (around 6 Å), and a long-range contact (larger than 7 Å, Figure 8B and 8D). The distributions of these distances are not the same in all replicas (Figure 8E), and neither are the distributions of the λ-coordinates associated with the doubly protonated state of HIS4 (Figure 8F). These differences suggest a lack of convergence of the conformational dynamics of the protein in constant pH MD. To test if the protonation state of HIS4 correlates with the distance, we performed standard MD of cardiotoxin V with HIS4 in the three different protonation states (Figure. S16). Because there is no clear correlation between the protonation state and the HIS4-TYR12 distance, we cannot conclude that the protonation states are coupled to the conformation of the pair, at least not directly. Instead, the differences between the replicas suggest a lack of sampling of these conformations. Because the local environment differs between the states, we speculate that this lack of conformational sampling also affects the λ-distributions. We therefore can only conclude that the sampling of these distances would require more than 100 ns to converge. Thus, even if the corrections to the torsion potentials overcome the convergence issues associated with sampling the intrinsic dihedral degrees of freedom in single amino acids, reaching converged sampling of the protonation states in proteins may still require longer time scales if the inherent conformational dynamics is too slow.

Nevertheless, we observe that compared to normal MD, constant pH MD can increase the sampling of the local conformational space of the protein. In Figures 8C and S17 we show that the HIS4 samples configurations more efficiently in constant pH simulations. Specifically, the hydrogen bond between PHE10 and the HIS4 δ-hydrogen is much more stable in normal MD with a fixed protonation of δ-nitrogen (Figure S17), whereas in constant pH MD, the HIS4 also samples configurations in which the H-bond is broken (Figure S17), in particular around pH = p*K*_a_, as evidenced by the distribution of the N–C_α_–C_β_–C_γ_ dihedral angle in Figure 8C. Thus, by keeping the protonation states flexible, constant pH MD facilitates the sampling of local conformations, which in turn may lead to faster convergence of the global conformational sampling.

## Conclusions

It is now possible to run accurate constant pH molecular dynamics simulations on time scales of normal simulations, for example, with the new implementation in the GROMACS package presented in the accompanying paper.^15^ Here, we have addressed the accuracy of constant pH simulation at longer time scales. We could demonstrate, on the basis of the CHARMM36m force field, that molecular force fields are not optimal for constant pH simulations because torsion barriers of titratable side chains are too high to reach convergence of the λ-coordinates associated with protonation. To overcome this sampling bottleneck, we proposed a systematic procedure to selectively reduce the barriers of the torsion potentials. In standard MD simulations, these modifications do not introduce noticeable artifacts but facilitate the convergence of side chain conformational sampling. Combined with the optimal fitting of *V*^MM^, these force field modifications constitute an essential preparation step for constant pH simulations.

The modifications of the CHARMM36m force field and the optimized parameters for the *∂V*^MM^/*∂λ* of correction potentials for Asp, Glu, His, Lys, and the C- and N-termini are available at https://gitlab.com/gromacs-constantph. Parameters for other force fields and residues will be made available there as well, once these are validated. We kindly ask the community to share with us also any parameters they may derive in their research, so that also these parameters can be made available.

The fork of GROMACS 2021 with constant pH MD implemented, as described here, is available for download free of charge from https://gitlab.com/gromacs-constantph/constantph. In addition to the source code, instructions on how to set up and perform MD simulations are available.

In addition to accurate parameters, it is also essential to keep the simulation system neutral during constant pH MD simulations. To achieve this, we introduced buffer particles with variable charges that dynamically compensate for the charge fluctuations of the titratable residues. To avoid that these buffers cluster, bind to the solute, disrupt hydrogen-bond networks, or penetrate into hydrophobic regions, we proposed a systematic parametrization procedure that can be used for any combination of force field and water model.

We expect the parametrization protocols proposed in this work to facilitate the application of constant pH MD not only within the user community of GROMACS but also of other MD programs as well. We also want to appeal to force field developers to take constant pH MD into consideration when developing their force fields.

## Supporting Information Available

Input files for MD simulations presented in this work (ZIP)
λ and angle distributions for Glu, His, Lys, and C- and N-termini; torsion corrections for Glu, His, and C-terminus; effects of force field modifications on dihedral dynamics and energy profiles; effects of fitting order on λ-distributions; conformations of HIS4 of cardiotoxin V (PDF)
