# Supplementary material for: Best Practices in Constant pH MD Simulations: Accuracy and Sampling
Source: J Chem Theory Comput. 2022 Sep 15;18(10):6134–47. doi: 10.1021/acs.jctc.2c00517 (PMC9558372; doi:10.1021/acs.jctc.2c00517)
Supplement: Supplementary file 2 — ct2c00517_si_002.pdf [file ct2c00517_si_002.pdf]

Supplementary Information: Best practices in constant pH MD simulations: accuracy and sampling

Pavel Buslaev,[*,†] Noora Aho,[†] Anton Jansen,[‡] Paul Bauer,[‡] Berk Hess,[*,‡] and Gerrit Groenhof[*,†]

†*Nanoscience Center and Department of Chemistry, University of Jyväskylä, Finland*

‡*Department of Applied Physics, Science for Life Laboratory, KTH Royal Institute of Technology, Stockholm, Sweden*

E-mail: pavel.i.buslaev@jyu.fi; hess@kth.se; gerrit.x.groenhof@jyu.fi

Contents

1 λ-distributions **3**

2 Torsion corrections **8**

3 Effect of FF modifications on standard MD **12**

4 Quality of correction potentials **15**

5 N-terminal loop of cardiotoxin V **21**

References **23**

1 λ-distributions

The distributions of the Glu, Lys, His, C-ter, and N-ter λ-coordinates obtained with the original and modified CHARMM36m force fields (Figures S1-5).

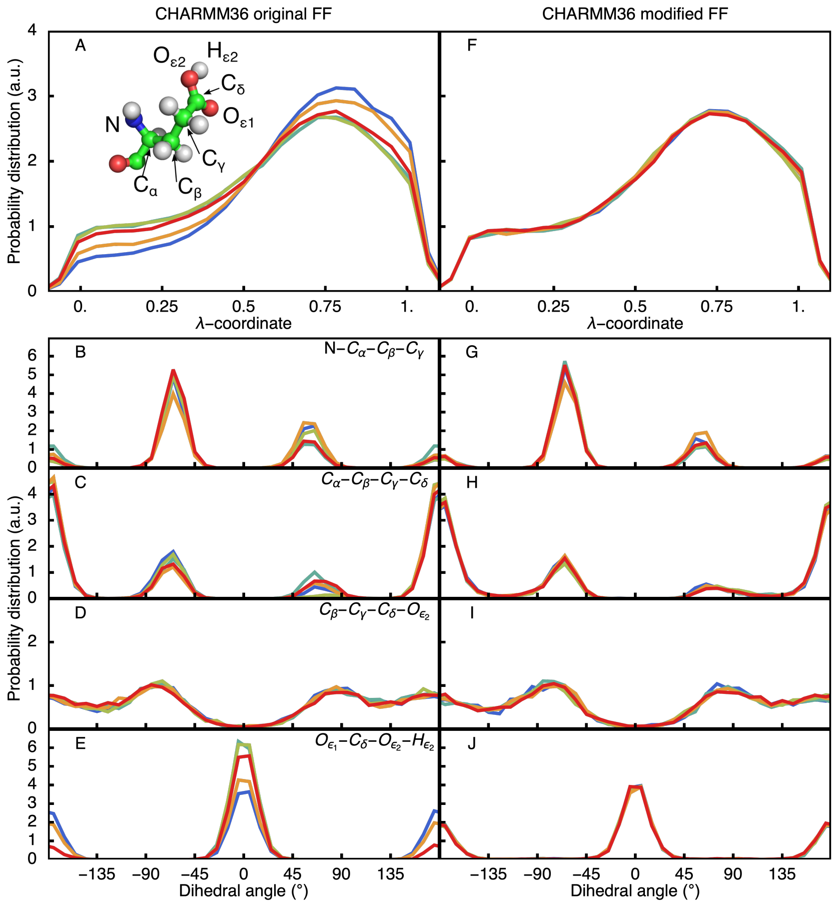

Figure S1: Distributions of Glu λ-coordinate (top row) and dihedral angles (rows 2-5). The left column shows the distributions for 3rd order V^{MM} fit obtained with original CHARMM36 force field. Different colors correspond to independent replicas. The distributions for λ-coordinate, as well as distributions for $N - C_\alpha - C_\beta - C_\gamma$, $C_\alpha - C_\beta - C_\gamma - C_\delta$ and $O_{\epsilon_1} - C_\delta - O_{\epsilon_2} - H_{\epsilon_2}$ are inconsistent between replicas. The right column shows the distributions for 3rd order V^{MM} fit with modified CHARMM36 force field, for which all the distributions are consistent.

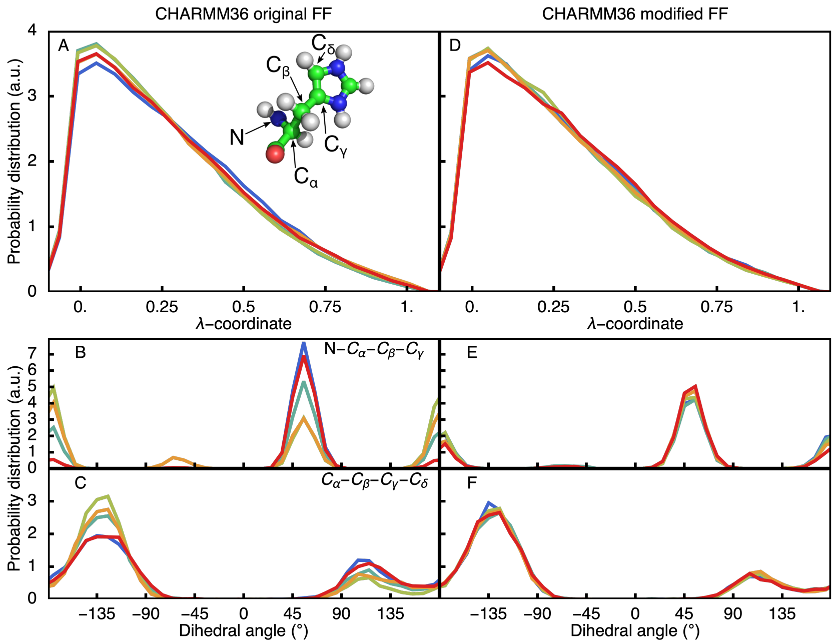

Figure S2: Distributions of His λ-coordinate (top row) and dihedral angles (rows 2-3). The left column shows the distributions for 3rd order V^{MM} fit obtained with original force field. Different colors correspond to independent replicas. The distributions for λ-coordinate, as well as distributions for $N - C_{\alpha} - C_{\beta} - C_{\gamma}$ and $C_{\alpha} - C_{\beta} - C_{\gamma} - C_{\delta_1}$ are inconsistent between replicas. The right column shows the distributions for 3rd order V^{MM} fit with modified CHARMM36 force field, for which all the distributions are consistent.

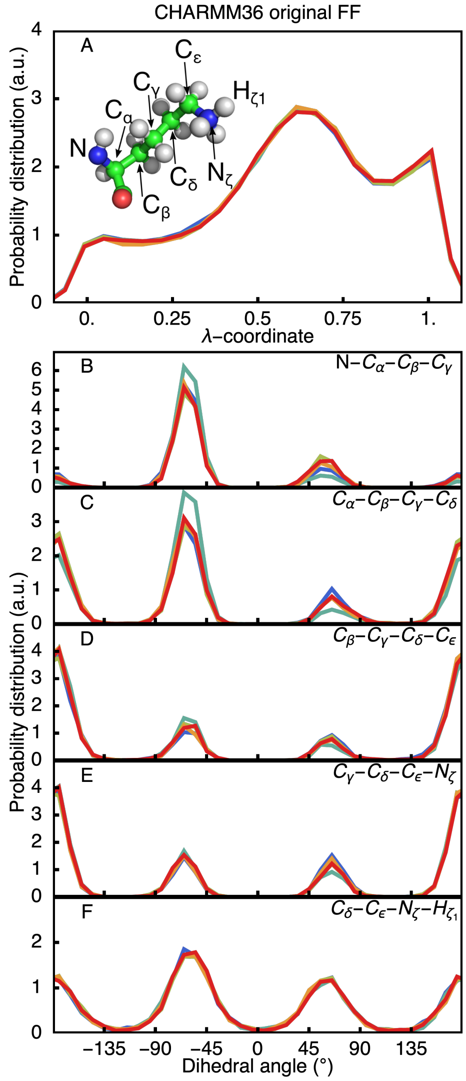

Figure S3: Distributions of Lys λ-coordinate (top row) and dihedral angles (rows 2-6). Different colors correspond to independent replicas. The distributions for 3rd order V^{MM} fit obtained with original force field are consistent, and thus no force field modifications are needed.

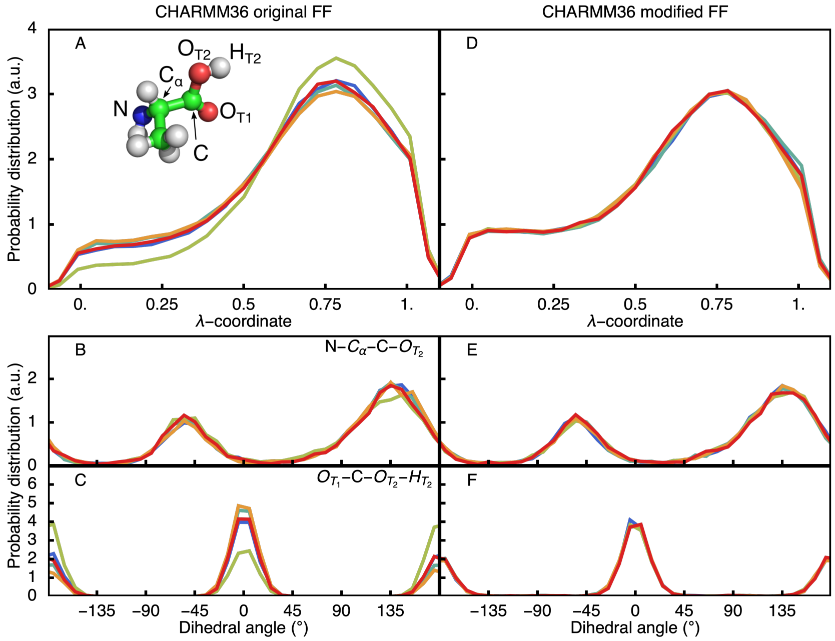

Figure S4: Distributions of C-terminus λ-coordinate (top row) and dihedral angles (rows 2-3). The left column shows the distributions for 3rd order V^{MM} fit obtained with original force field. Different colors correspond to independent replicas. The distributions for λ-coordinate, as well as distribution for $O_{\mathrm{T}_1} - C - O_{\mathrm{T}_2} - O_{\mathrm{T}_2}$ dihedral are inconsistent. The right column shows the distributions for 3rd order V^{MM} fit with modified CHARMM36 force field, for which all the distributions are consistent.

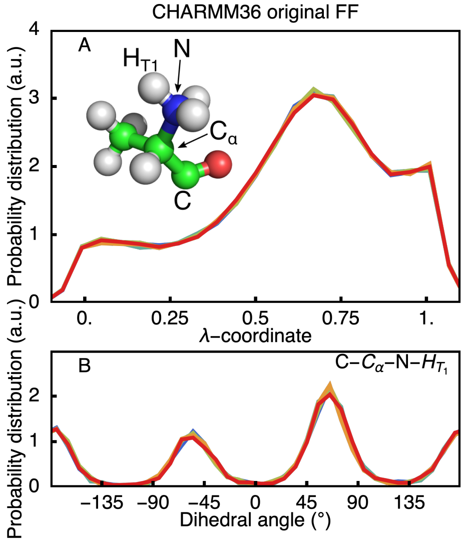

Figure S5: Distributions of N-terminus λ-coordinate (top row) and dihedral angles (bottom row). Different colors correspond to independent replicas. The distributions for 3rd order V^{MM} fit obtained with original force field are consistent, and thus no force field modifications are needed.

2 Torsion corrections

The corrections applied to torsions of GLU, HIS, and C-terminal (Figures S6-8).

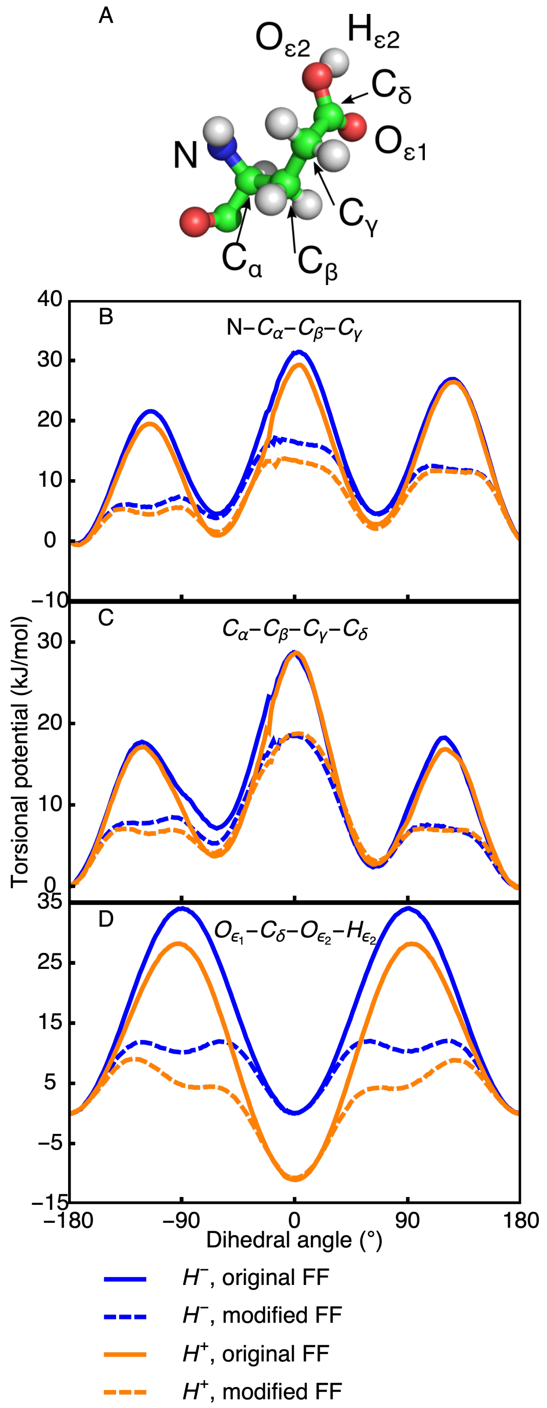

Figure S6: Modification of the Glu torsional barriers. Glutamic acid and its atomic nomenclature is shown in the top panel. Bottom rows show original and modified torsional barriers of Glu for both protonated and deprotonated states.

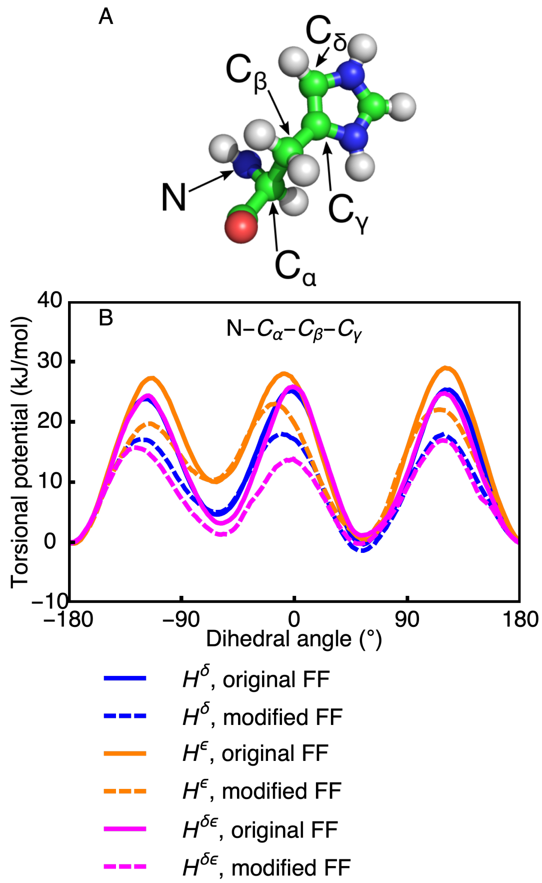

Figure S7: Modification of the His torsional barriers. Histidine and its atomic nomenclature is shown in the top panel. Bottom panel show original and modified torsional barriers of His for all possible protonation states.

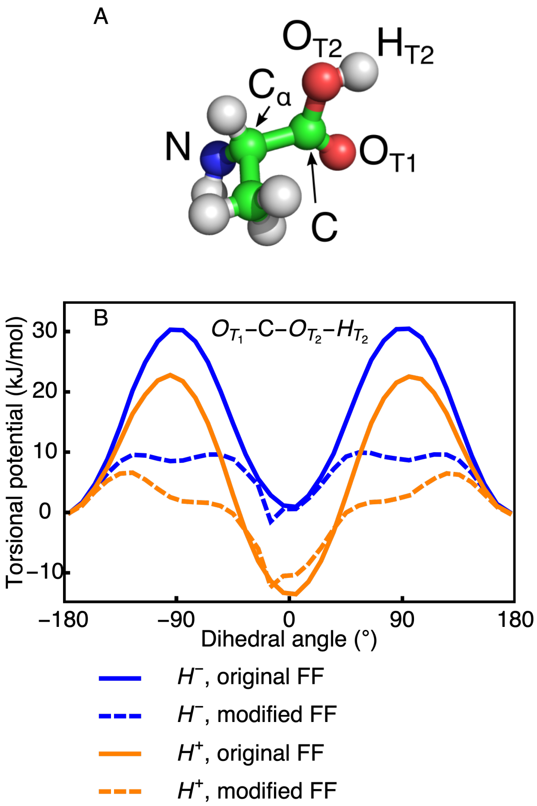

Figure S8: Modification of the C-terminus torsional barriers. C-terminus and its atomic nomenclature is shown in the top panel. Bottom panel show original and modified torsional barriers of the C-terminus for both protonated and deprotonated states.

3 Effect of FF modifications on standard MD

Comparison of potential energy profiles of Asp C_α-C_β obtained with MP2/6-31+G* level of theory using Firefly QC package,[1] which is partially based on the GAMESS (US)[2] source code with MM profiles obtained with original and modified CHARMM36m force field (Figure S9).[3,4] Distributions and dynamics of Asp torsions obtained in 100 ns standard MD simulations (Figure S10).

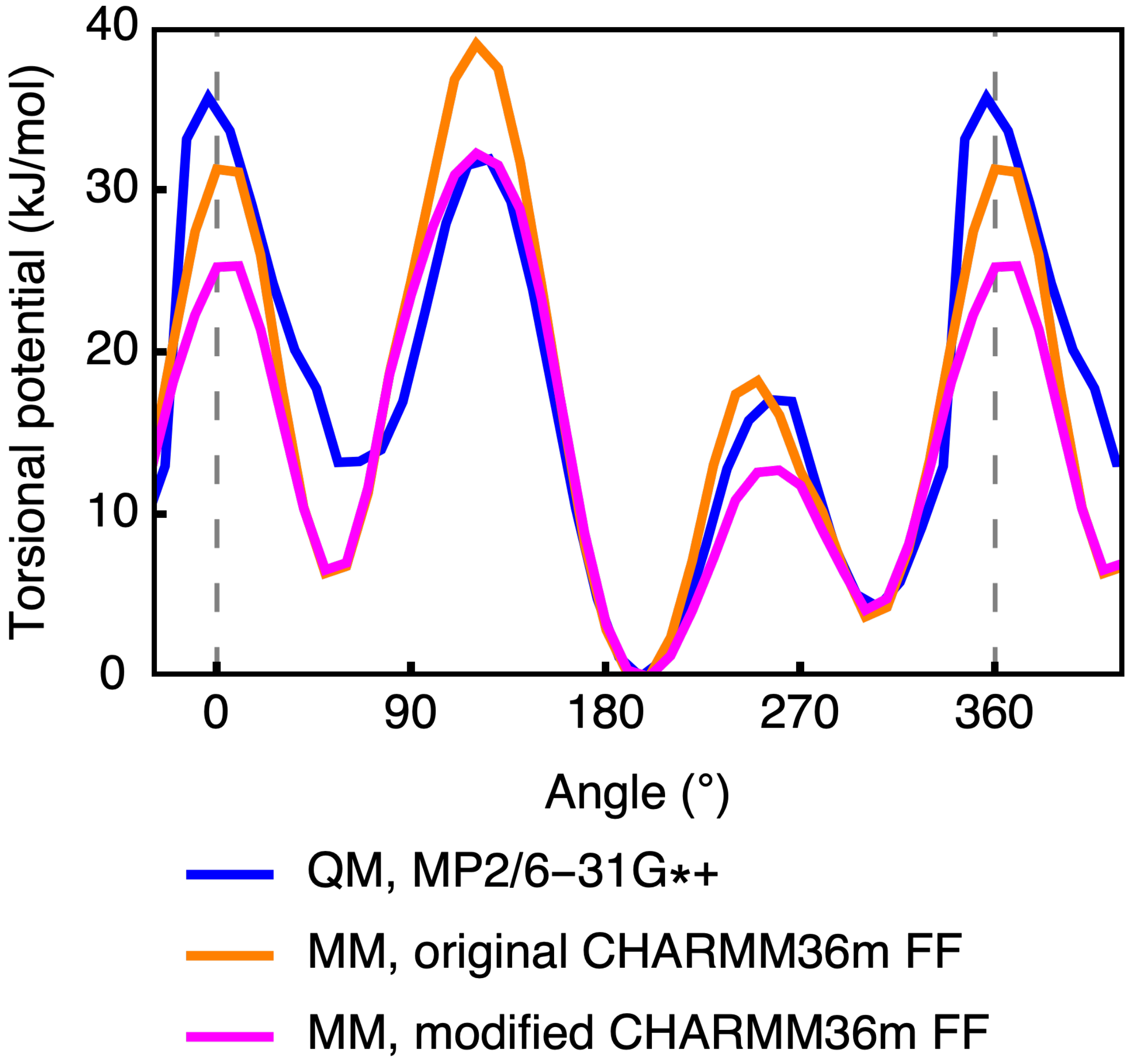

Figure S9: Potential energy profile of N-C_α-C_β-C_γ dihedral. Note that we show profiles from -30° to 420°. For the modified force field the root mean square deviation from the QM profile is reduced from 4.0 kJ/mol for the original force field to 3.5 kJ/mol for the modified force field.

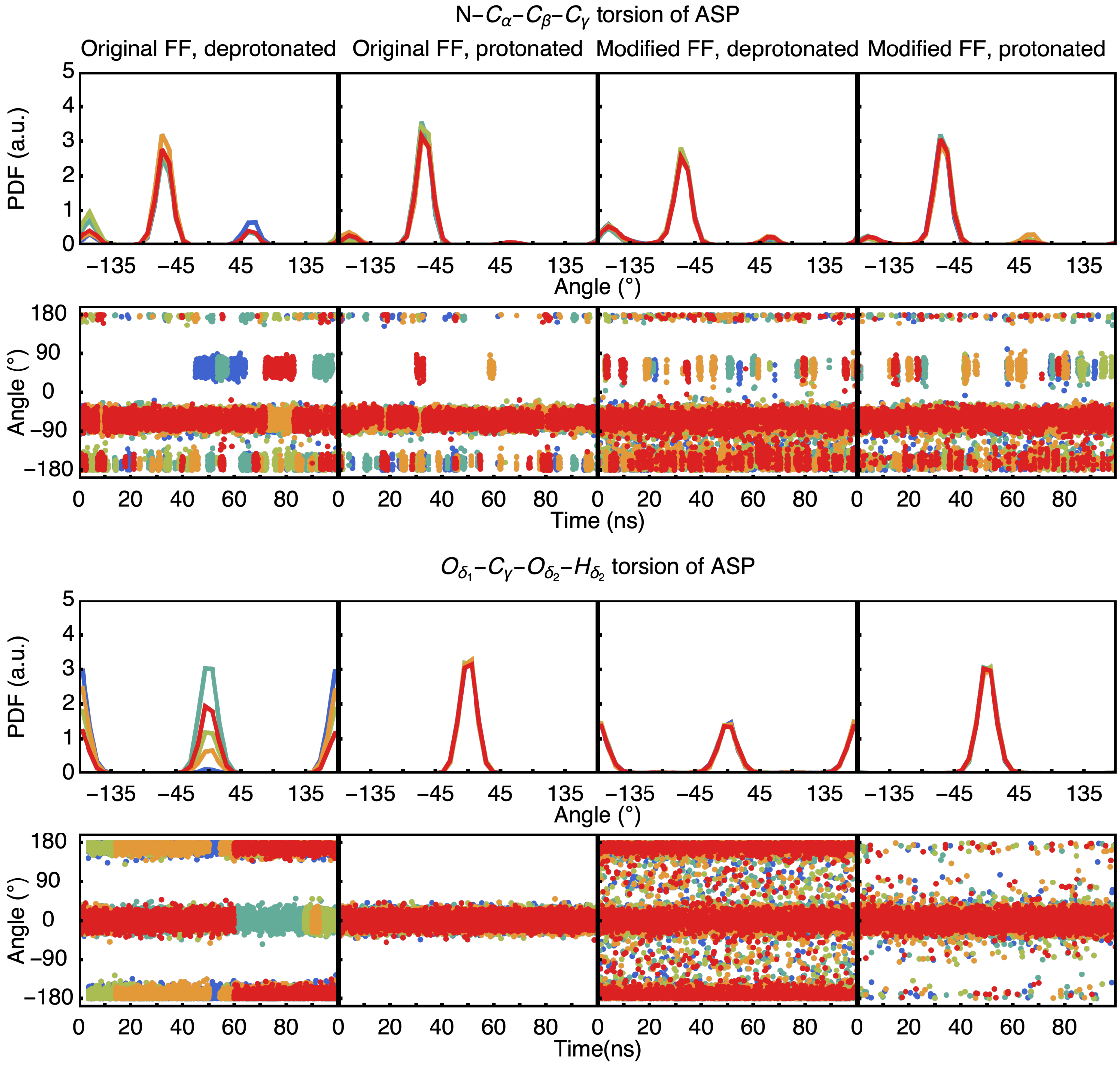

Figure S10: Distributions of torsional angles and trajectories for the torsions that were modified for Asp, obtained using standard MD. Different colors correspond to different replicas.

4 Quality of correction potentials

Fitting error and λ-distributions for low and high fitting orders for Glu, Lys, His, C- and N-termini (Figures S11-14).

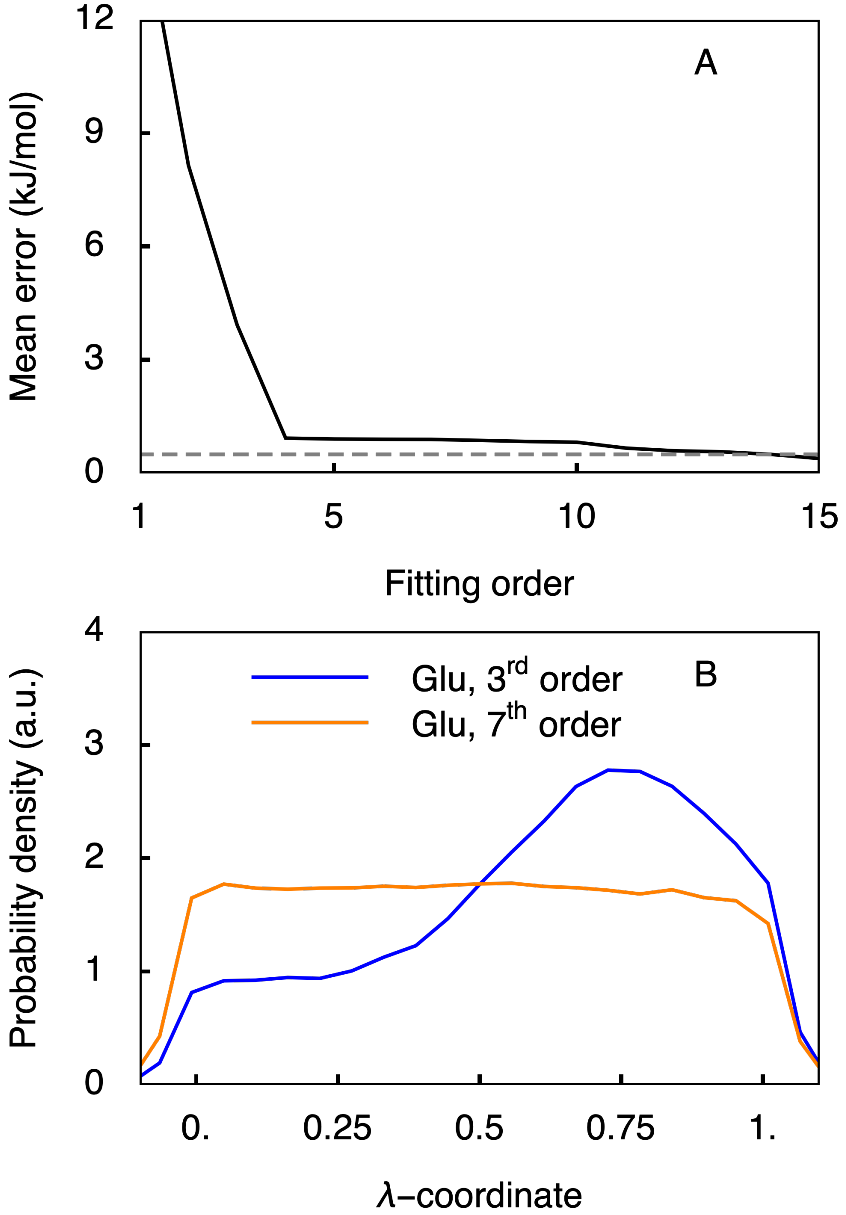

Figure S11: Fitting quality for Glu. The top panel shows the fitting error as a function of fitting order (black line). The gray dashed line shows the average error of calculated $\frac{\partial V}{\partial \lambda}$. On the bottom panel, the λ-distributions for the 3rd and 7th order fittings are shown. While for the low order fitting the distribution is significantly rugged, it gets almost ideally flat at 7th order.

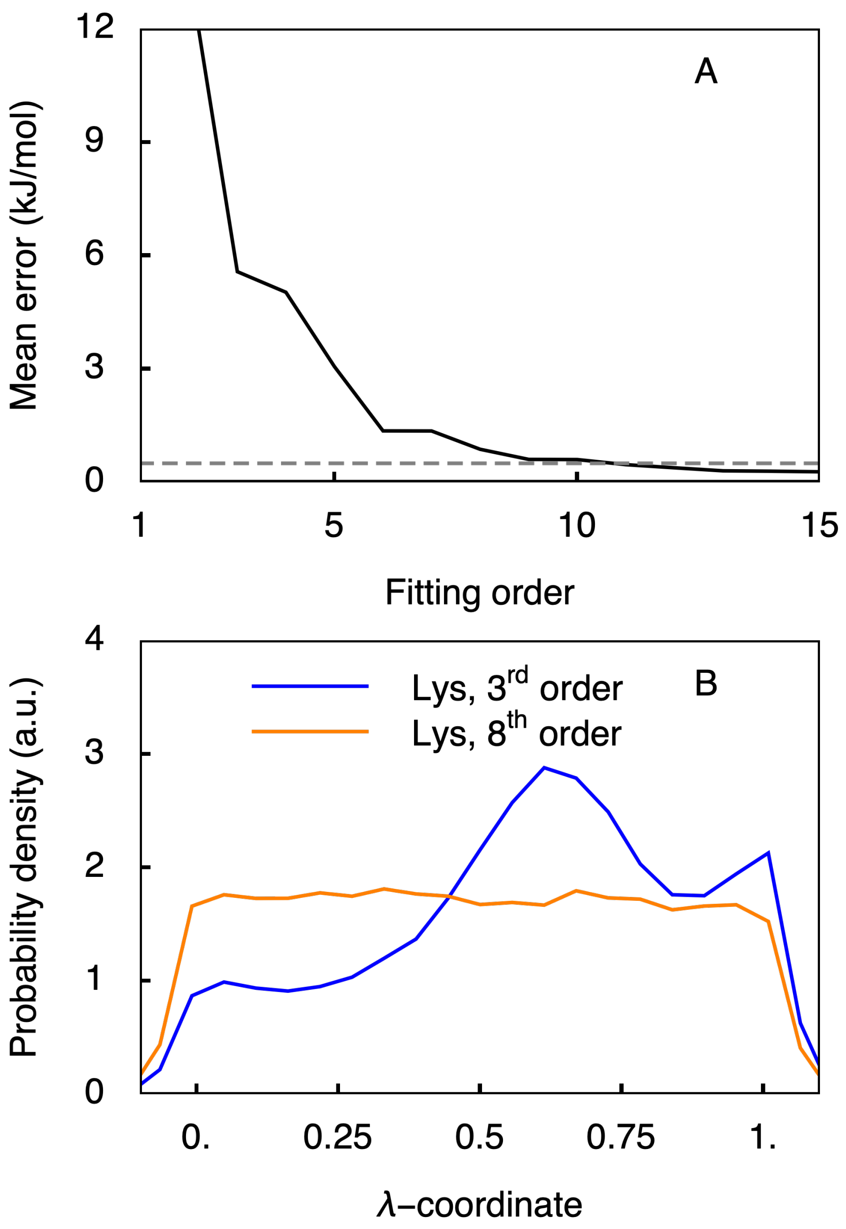

Figure S12: Fitting quality for Lys. The top panel shows the fitting error as a function of fitting order (black line). The gray dashed line shows the average error of calculated $\frac{\partial V}{\partial \lambda}$. On the bottom panel, the λ-distributions for the 3rd and 8th order fittings are shown. While for the low order fitting the distribution is significantly rugged, it gets almost ideally flat at 8th order.

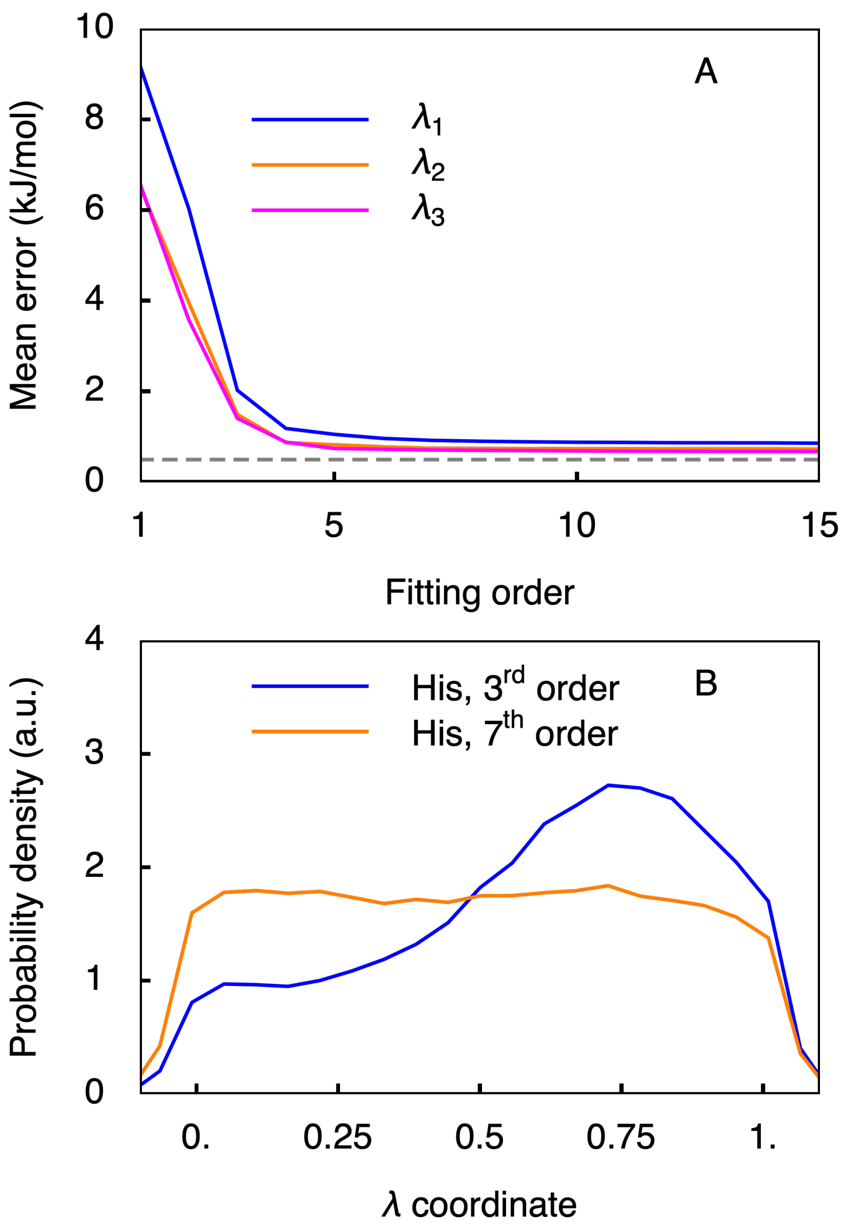

Figure S13: Fitting quality for His. The top panel shows the fitting error as a function of fitting order (black line). The gray dashed line shows the average error of calculated $\frac{\partial V}{\partial \lambda}$. On the bottom panel, the λ-distributions for the 3rd and 7th order fittings are shown. While for the low order fitting the distribution is significantly rugged, it gets almost ideally flat at 7th order.

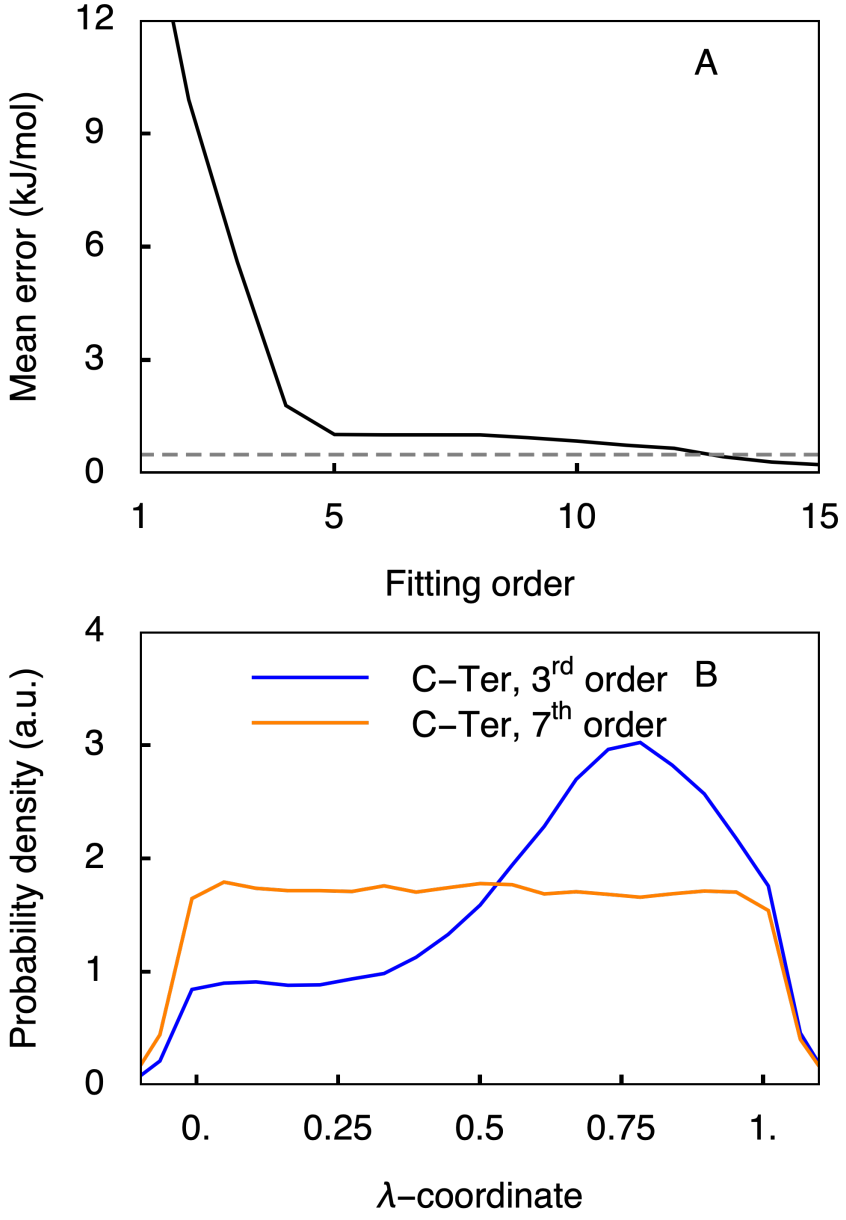

Figure S14: Fitting quality for C-terminus. The top panel shows the fitting error as a function of fitting order (black line). The gray dashed line shows the average error of calculated $\frac{\partial V}{\partial \lambda}$. On the bottom panel, the λ-distributions for the 3rd and 7th order fittings are shown. While for the low order fitting the distribution is significantly rugged, it gets almost ideally flat at 7th order.

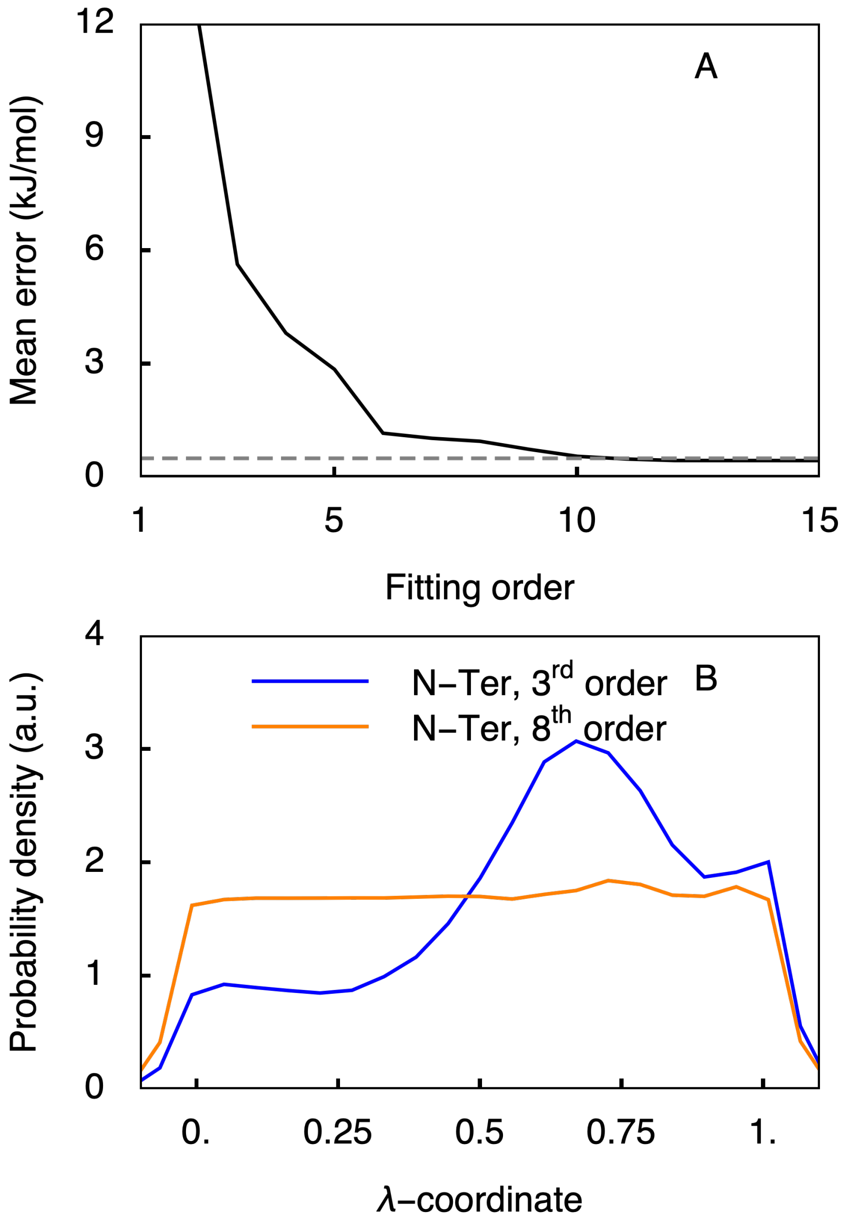

Figure S15: Fitting quality for N-terminus. The top panel shows the fitting error as a function of fitting order (black line). The gray dashed line shows the average error of calculated $\frac{\partial V}{\partial \lambda}$. On the bottom panel, the λ-distributions for the 8rd and 7th order fittings are shown. While for the low order fitting the distribution is significantly rugged, it gets almost ideally flat at 8th order.

5 N-terminal loop of cardiotoxin V

HIS-4-TYR-12 distance distributions obtained in 100 ns standard MD simulations with original CHARMMM36m FF[4] and three fixed protonation states of Histidine (Figure S16). Distribution of the distance between HIS-4 δ-hydrogen and PHE-10 backbone oxygen in constant pH and standard MD simulations (Figure S17).

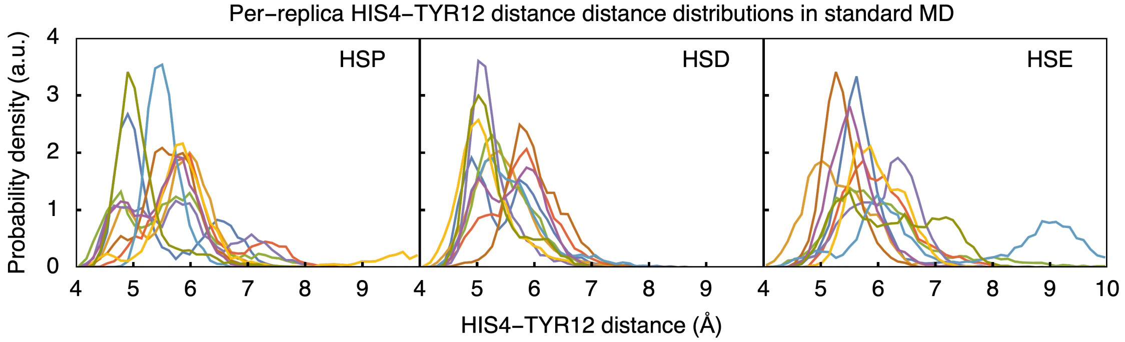

Figure S16: Distributions of distance between HIS-4 and TYR-12 of cardiotoxin V obtained in 10 replicas of 100 ns standard MD simulations with HIS in three different protonation states. Different colors correspond to different replicas.

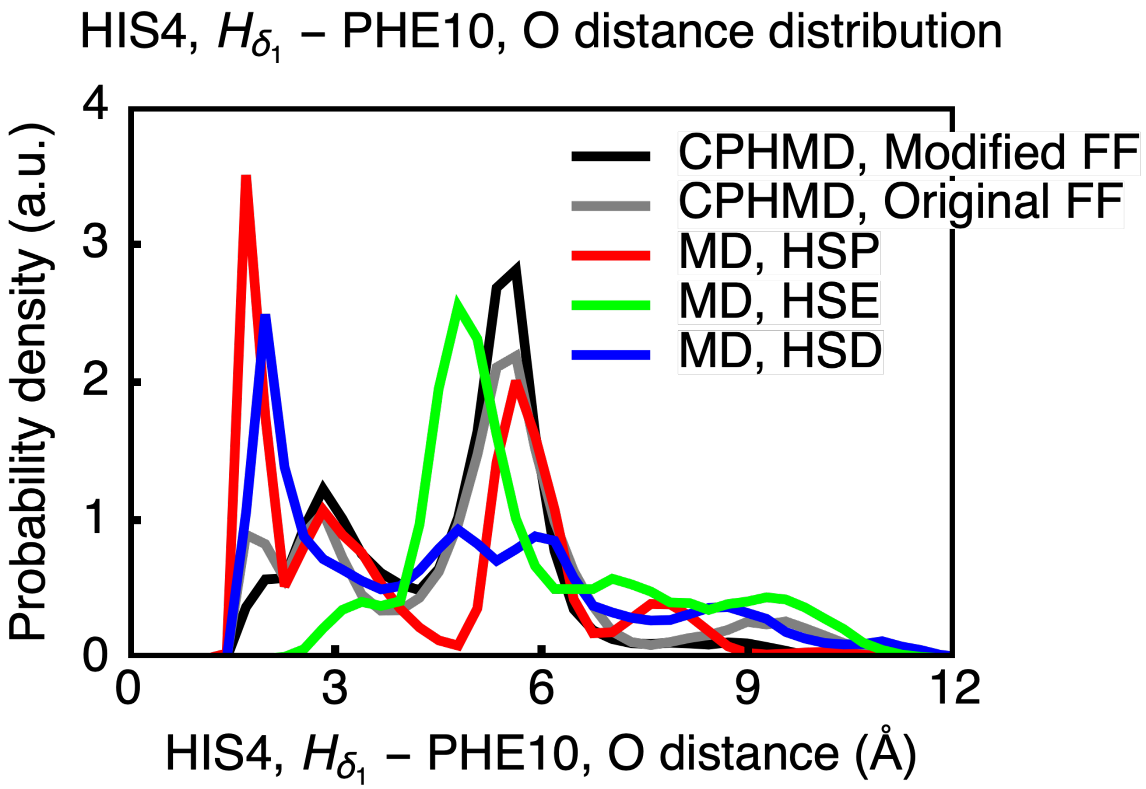

Figure S17: Distribution of distance between HIS-4 δ hydrogen and PHE-10 backbone oxygen obtained in constant pH MD with original (gray) and modified (black) CHARMM36 force fields, and with standard MD for three fixed protonation states of HIS-4 (HSP - red, HSD - green, HSE - blue). Distribution are calculated from all 10 replicas of 100 ns in each case.

References

(1) Granovsky, A. A. Firefly version 7.1. G, accessed by 01.07.2022. *http://classic.chem.msu.su/gran/gamess/index.html* **2012**,

(2) Schmidt, M. W.; Baldridge, K. K.; Boatz, J. A.; Elbert, S. T.; Gordon, M. S.; Jensen, J. H.; Koseki, S.; Matsunaga, N.; Nguyen, K. A.; Su, S.; Windus, T. L.; Dupuis, M.; Montgomery Jr, J. A. General atomic and molecular electronic structure system. *Journal of computational chemistry* **1993**, *14*, 1347–1363.

(3) Best, R. B.; Zhu, X.; Shim, J.; Lopes, P. E.; Mittal, J.; Feig, M.; MacKerell Jr, A. D. Optimization of the additive CHARMM all-atom protein force field targeting improved sampling of the backbone ϕ, ψ and side-chain χ1 and χ2 dihedral angles. *Journal of chemical theory and computation* **2012**, *8*, 3257–3273.

(4) Huang, J.; Rauscher, S.; Nawrocki, G.; Ran, T.; Feig, M.; De Groot, B. L.; Grubmüller, H.; MacKerell, A. D. CHARMM36m: an improved force field for folded and intrinsically disordered proteins. *Nature methods* **2017**, *14*, 71–73.
